# Reliable ligand discrimination in stochastic multistep kinetic proofreading: First passage time vs. product counting strategies

**DOI:** 10.1371/journal.pcbi.1012183

**Published:** 2024-06-10

**Authors:** Xiangting Li, Tom Chou

**Affiliations:** 1 Department of Computational Medicine, University of California, Los Angeles, California, United States of America; 2 Department of Mathematics, University of California, Los Angeles, California, United States of America; Rutgers University, UNITED STATES

## Abstract

Cellular signaling, crucial for biological processes like immune response and homeostasis, relies on specificity and fidelity in signal transduction to accurately respond to stimuli amidst biological noise. Kinetic proofreading (KPR) is a key mechanism enhancing signaling specificity through time-delayed steps, although its effectiveness is debated due to intrinsic noise potentially reducing signal fidelity. In this study, we reformulate the theory of kinetic proofreading (KPR) by convolving multiple intermediate states into a single state and then define an overall “processing” time required to traverse these states. This simplification allows us to succinctly describe kinetic proofreading in terms of a single waiting time parameter, facilitating a more direct evaluation and comparison of KPR performance across different biological contexts such as DNA replication and T cell receptor (TCR) signaling. We find that loss of fidelity for longer proofreading steps relies on the specific strategy of information extraction and show that in the first-passage time (FPT) discrimination strategy, longer proofreading steps can exponentially improve the accuracy of KPR at the cost of speed. Thus, KPR can still be an effective discrimination mechanism in the high noise regime. However, in a product concentration-based discrimination strategy, longer proofreading steps do not necessarily lead to an increase in performance. However, by introducing activation thresholds on product concentrations, can we decompose the product-based strategy into a series of FPT-based strategies to better resolve the subtleties of KPR-mediated product discrimination. Our findings underscore the importance of understanding KPR in the context of how information is extracted and processed in the cell.

## Author summary

Kinetic proofreading (KPR) is mechanism often employed by cells to enhance specificity of ligand-receptor. However, the performance of kinetic proofreading may be hampered by noise and a low signal-to-noise ratio. By consolidating multiple kinetic proofreading steps into a single state and assigning an associated waiting, or “processing time,” we developed an analytic approach to quantify the performance of KPR in different biological contexts. Despite a trade-off between speed and accuracy inherent to a first-passage time KPR strategy, we show that a signaling molecule-based discrimination strategy can enhance the performance benefits of KPR. We further decompose the product-based discrimination strategy into a set of first-passage times to different thresholds of signaling molecules produced. Through this decomposition, we find that a threshold that adjusts dynamically throughout the recognition process depends on the duration of the process. We propose that this more nuanced product-based KPR-mediated recognition process can be realized biologically. The precise structural basis for a dynamic threshold merits further experimental exploration, as it may hold significant implications for understanding biological mechanisms of information transmission at a molecular level.

## Introduction

Various cellular processes require a high degree of specificity in order to function properly, including DNA replication, gene expression, and cellular signaling. The degree of specificity observed is often hard to justify by a simple binding-affinity argument, the specificity of which is proportional to exp(−ΔΔ*G*/*RT*), where ΔΔ*G* is the difference in free energy between the correct and incorrect ligands [1]. For example, the estimated error probability per nucleotide in DNA replication is estimated to be 10^−9^ [2], but the net free energy difference between mismatched and matched base pairs is only 0.5 kcal/mol [3], suggesting the theoretical error rate would be ∼ 10^−3^. Similarly, T cells need to specifically distinguish self-antigens from mutant self-antigens, also known as neoantigens, which can differ by only one or a few amino-acids [4].

**Fig 1:**
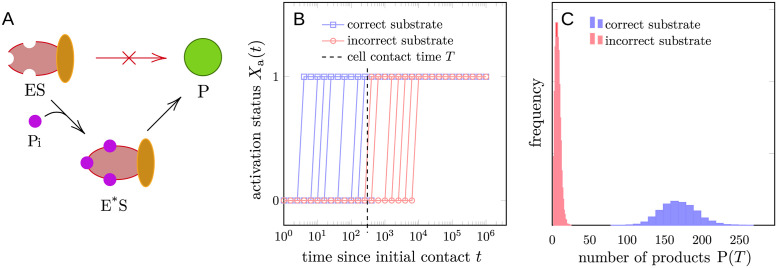
Schematic of the KPR process and different strategies of interpreting the output. (A) The complex of enzyme and substrate ES alone cannot produce the final product P. It has to undergo a number of proofreading steps, represented here by phosphorylation (Pi), before the activated state E*S can produce the final product. (B) The FPT-based discrimination strategy, simply reaching the activated state E*S is interpreted as the output. *X*_a_(*t*) = 0 if the system has not reached the activated state by time *t*, and *X*_a_(*t*) = 1 otherwise. At *t* = 0, the reaction starts with the enzyme in the free state E. The dashed vertical line represents the termination of entire process, *e.g.*, the time *T* at which the T cell and antigen presenting cell (APC) separate. *X*_*a*_(*t*) = 1 for some *t* < *T* is interpreted as a positive response. (C) The product-based discrimination strategy. In this strategy, the number of product molecules P(*T*) produced within a given time *T* is interpreted as the output. Due to the intrinsic noise, the number of product molecules is a random variable and their distribution is shown for correct and incorrect ligands.

Kinetic proofreading (KPR) [1, 5, 6] typically denotes a chemical reaction mechanism that can significantly increase the specificity towards a desired ligand against competing ligands. In the KPR context, “proofreading” is accomplished by introducing additional irreversible, energy-consuming kinetic steps which individually may not distinguish desired ligands from undesired ones. However, these steps impart a delay to final product release allowing for “resetting” of the process and an overall lower final error rate (a multistep KPR mechanism is illustrated in Fig 1A below and mathematical details are discussed in the Materials and methods).

First proposed by Hopfield to explain the high specificity of DNA/protein synthesis [1], the KPR mechanisms have been invoked to explain other biological processes such as T cell receptor (TCR) signaling [6] and microtubule growth [7]. These first treatments of KPR described it within the steady-state limit of deterministic mass-action models, comparing the steady-state fluxes of the correct and incorrect product formation. Reactions inside the cell, however, are often between small numbers of molecules and are thus stochastic. Stochastic aspects of KPR have also been considered, emphasizing the statistics of first passage times (FPT) to product formation [8–10].

Recently, Kirby and Zilman reported that adding more kinetic proofreading steps almost always decreases the signal-to-noise ratio (SNR) defined by the ratio of the mean to the standard deviation of the output signal (the number of signaling molecules produced within a time period), suggesting that KPR is not an optimal strategy for information processing due to noise [11]. However, TCR signaling and T cell activation, the context that Kirby and Zilman describe, is a highly specific process that does involve multiple kinetic proofreading steps, but with adaptive variants of KPR used to model TCR signaling [12–16].

In this paper, we reconcile the apparent contradiction between the high specificity of TCR signaling and the low SNR of a longer-chain KPR process. The key theoretical insight involves convolving the multiple intermediate irreversible steps into a single equivalent state in which the system stays for time *τ*. Instead of explicitly treating a series of sequential states, we define a single, equivalent waiting time or “processing” time *τ* which may be a random variable. In this work, we will primarily take *τ* to be sharply peaked around its mean value, *i.e.*, a *δ*-function distribution, resulting in a “deterministic” processing time *τ*. A similar quantity was proposed in the context of kinetic segregation in TCR signaling [17]. In our subsequent analyses, we also need to define a contact time *T* that represents the duration in which the overall process can occur, *e.g.*, a cell-cell contact time. Values of *T* may also follow a modeled distribution. This simple reduction reveals intriguing insights, allows us to analytically and systematically explore different biological contexts of KPR, and provides an easier framework on which to test different metrics of KPR performance. We will primarily focus on the TCR recognition process, but we will also provide a short discussion on the simpler DNA replication problem, to illustrate that our deterministic processing time approach precisely captures the essence of KPR.

We show that the apparent contradiction arises from different strategies of determining whether a final output is correct. In the FPT-based scenario for both DNA replication and TCR signaling, arriving at an activated state or a “product” state within a given time is interpreted as the output, as illustrated in Fig 1B. A longer processing time *τ* can exponentially improve the accuracy of KPR at the cost of speed. The trade-off between speed and accuracy has been reported in experimental studies [18–20] recently emphasized in Xiao and Galstyan [21].

In an alternative strategy for TCR signaling implicitly used by Kirby and Zilman [11], the maximum SNR, or mutual information between the input and output occurs at just two proofreading steps, with additional processing steps decreasing the performance of KPR. Here, the implicit discrimination strategy detects the number of products (*e.g.*, signaling molecules that lead to downstream processes) generated within a finite time *T* without explicitly resolving the final response of the cell, as is illustrated in Fig 1C. While Kirby and Zilman also considered a FPT-based strategy to support use of their metric and their conclusions, a recent communication by Xiao and Galstyan [21] suggested that the previous simulation of the FPT-based strategy was not correctly implemented.

Mutual information has been used in recent studies to quantify the information flow in cellular decision-making processes [22–24]. Here, we introduce mutual information and channel capacity in order to compare the performance of the two strategies (FPT to a target state and product counting) on equal footing. We also construct a decomposition of the product-based strategy into a series of FPT-based strategies with different product molecule thresholds and conclude that the product detection strategy is equivalent to a strategy that dynamically adjusts the threshold according to the duration of the process. This dynamic thresholding strategy can be shown to be more robust to fluctuations over the duration of the reaction. The effectiveness of this strategy can be attributed to an additional layer of proofreading.

Our analysis and findings present a unified framework for analyzing KPR under different biological scenarios. We also highlight the importance of understanding how different strategies of information extraction can affect the performance and parameter tuning of KPR.

## Materials and methods

In this section, we first describe the general model of KPR and then apply it to two specific biological contexts, DNA replication and TCR signaling.

### Model settings

In the conventional setting of KPR, the complex E^(0)^S composed of receptor E and a “correct” substrate (or ligand) S forms and dissociates with binding and unbinding rates *k*_±1_. A complex with the “incorrect” ligand forms and dissociates with rates $k_{\pm 1}^{\prime }$. In all of our subsequent analyses and approximations, we will assume constant availability of substrate and define *k*_1_ as a first-order reaction rate. Since an incorrect ligand unbinds faster than the correct, stronger binding one, we will take $k_{-1}^{\prime }>k_{-1}$. Both types of complexes can undergo multiple nonequilibrium transitions or proofreading steps (*e.g.*, sequential phosphorylation) traversing internal states (E^(0)^S, …, E^(*m*−1)^S) before the final product P can be released or produced by the fully activated state E*S.

**Fig 2:**
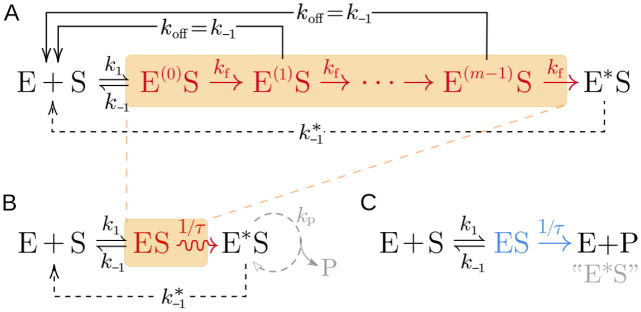
Reaction schemes of different descriptions of the simple kinetic proofreading process. (A) The conventional description of KPR explicitly incorporating multiple proofreading steps. (B) A reduced representation of KPR in which multiple driven steps are lumped in a single proofreading step. Additionally, the state E*S can be taken to be a final “product” state E + P, in which case, there is no disassembly (marked by dashed lines) and $k_{-1}^{*}\equiv 0$. However, one may be interested in products P that are constitutively produced (at rate *k*_p_) by the E*S state (gray). This latter scenario can describe, *e.g.*, TCR-mediated T cell activation. (C) For comparison, we show the classical Michaelis-Menten reaction scheme in which the “product” state E + P can be identified as an activated complex E*S. The Michaelis-Menten kinetics implicitly assumes values of *τ* are exponentially distributed. An internal proofreading process leads to a nonexponentially distributed *τ* and equivalence to the scheme in (B) and describes the FPT-based DNA replication setting (without any additional constitutive product formation). In our setting, substrate concentrations are held fixed, and *k*_1_ will be defined as a first-order reaction rate with physical units of 1/time.

Each internal state of the complex can dissociate with rate *k*_off_ or proceed to the next step with rate *k*_f_, as shown in Fig 2A. To simplify our subsequent analysis, we set *k*_off_ = k_−1_ as in [1, 6, 10, 11], effectively assuming that the phosphorylation of the enzyme does not allosterically alter the binding affinity between the enzyme and substrate. Additionally, for an *m*-step proofreading process, we set *k*_f_ = *m*/*τ*. Under this scaling, the waiting time to reach the final activated state is a sum of *m* independent exponential random variables with rate *m*/*τ*. In other words, the waiting time to reach the final activated state follows an Erlang distribution with shape parameter *m* and rate *m*/*τ*. The mean waiting time is kept fixed at *τ* as *m* increases, while the variance *τ*^2^/*m* decreases as *m* increases. In the many-step limit (*m* → ∞), the waiting time to reach the final activated state converges to a deterministic value *τ* due to the strong law of large numbers. A general situation in which the activation steps are partially reversible is discussed in [9]. We can thus simplify the reaction diagram in Fig 2A by lumping the internal states (E^(0)^S, …, E^(*m*−1)^S) into a single state ES as shown in Fig 2B. Because activation and disassembly of each stage are independent, the transition of the aggregated state ES to the dissociated state E + S is still Markovian with the same rate *k*_−1_. The master equation of the simplified model and its relation to the original multi-step model are discussed in Appendix A1 of S1 Text.

As shown in Fig 2C, our simplified model has a similar structure to the classical Michaelis-Menten reaction scheme but implicitly captures a crucial ingredient of kinetic proofreading. In the Michaelis-Menten scheme, the waiting time *τ* in the complex state ES before converting to product is exponentially distributed. In the KPR scheme, the waiting time *τ* in the state ES before converting to the activated state E*S is assumed to be non-exponentially distributed. An exponentially distributed waiting time reflects a memoryless process in which the evolution of the system depends only on the current state. This memoryless property is the defining feature of a Markov process. By contrast, memory in the KPR reaction process results in a non-exponential distribution of the processing time *τ*, such as the Erlang distribution associated with an *m*-step processing chain described above. In the simplification of the KPR we are considering, the processing time is fixed to the value *τ*, *i.e.*, its distribution is a Dirac-delta function at *τ* (the *m* → ∞ limit of an Erlang distribution). In this case, the waiting time of activation does not depend only on the current state, but also on the time elapsed since the initial formation of the complex, which happened in the past. This non-Markovian step acts as a memory of the system or a clock that keeps track of the time elapsed since the initial formation of the complex, and can be achieved by, *e.g.*, tracking the phosphorylation state of the complex ES. The biological context in which KPR operates will determine the most informative and realistic KPR model structure and the most appropriate performance metric.

Extensions to dynamic KPR schemes, including adaptive KPR models [12–16], force-dependent signaling through catch bonds [25, 26], and KPR in the presence of a spatial gradient [27], have been introduced in the literature. While canonical KPR models [1, 6, 10, 11] assume a homogeneous state independent activation rate *k*_f_ and disassembly rate *k*_−1_ = *k*_off_, more general models can incorporate heterogeneity in the activation or unbinding rates across the processing steps. In such scenarios, we can still gain quantitative insight by using a mean-field approximation in which our deterministic processing time is taken to be the mean unbinding time. For example, in the multistep KPR model, the unbinding rate *k*_−1_ of the initial complex may differ from the unbinding rate *k*_off_ of the intermediate complexes. But since the complex spends most of the time in the intermediate states when the number of steps *m* is large, the contribution of the initial complex unbinding rate *k*_−1_ to the overall disassembly rate is negligible. As a result, the assumption *k*_−1_ = *k*_off_ can be interpreted as a mean-field approximation in which we substitute the disassembly rate of the intermediate complexes for the unbinding rate of the initial complex. Additionally, depending on the specific pattern of the activation rate and the disassembly rate, our approach can be extended by lumping together connected states with similar activation or disassembly rates. Within each lumped state, the internal “microstates” carry similar activation and disassembly rates. Thus, our overall approach can also provide analytical and quantitative understanding of these more general KPR models. Lastly, the key idea of our method is to convolve multiple waiting times into a single, non-exponential waiting time, which carries “memory” of the past states. This idea can also be applied to the unbinding process when the assumption *k*_−1_ = *k*_off_ fails, although in that case, analytical results may be more difficult to obtain. However, our method can still provide a systematic way to quantitatively and numerically understand how the “memory” of the past states affects the overall performance of KPR.

### DNA replication setting

**Fig 3:**
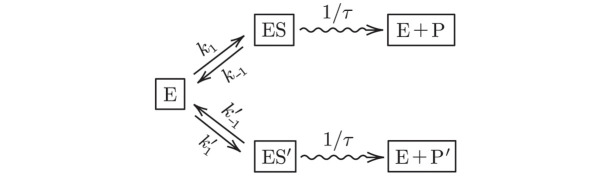
The simplified model of KPR in DNA replication with only one enzyme. Here, the enzyme (*e.g.*, DNA polymerase) has three states, namely, free (E), bound to correct substrate (ES), and bound to incorrect substrate (ES′). The transition rates between these states are specified in the model. When the enzyme is bound to substrate, it produces the product (P or P′) after a waiting time *τ*.

For completeness and to define a reference case, we consider the DNA replication scenario, specifically a single nucleotide incorporation step catalyzed by DNA polymerase (E). We track the system starting from an initial state where the enzyme is free. A correct substrate S refers to the complementary nucleotide to the template strand, while an incorrect substrate S′ refers to the other three nucleotides. The enzyme can bind to either substrate with rates *k*_1_ and $k_{1}^{\prime }$, respectively. The enzyme can also unbind from either substrate with rates *k*_−1_ and $k_{-1}^{\prime }$. This two-branch reaction scheme is given by

$$\begin{array}{c} \begin{array}{c} \text{E}+\text{S}\underset{k_{-1}}{\overset{k_{1}}{⇌}}\text{ES}\overset{1/\tau }{⇝}\text{E}+\text{P},\;\text{E}+{\text{S}}^{\prime }\underset{k_{-1}^{\prime }}{\overset{k_{1}^{\prime }}{⇌}}\text{E}{\text{S}}^{\prime }\overset{1/\tau }{⇝}\text{E}+{\text{P}}^{\prime }, \end{array} \end{array}$$ (1)

where P and P′ denote the correct and incorrect products. We track the system until either one of the products is produced, allowing repeated binding and unbinding of the enzyme to the substrates. While there can be multiple replication forks in a cell, we focus on a single DNA polymerase in this study. Thus, we can represent the stochastic system by a simplified stochastic process with described by three transient states indicating the status of the DNA polymerase; namely, unbound polymerase (E), polymerase bound to correct substrate (ES), and polymerase bound to incorrect substrate (ES′). There are also two absorbing states, namely, correct product (E + P) and incorrect product (E + P′). This simplified stochastic scheme is shown in Fig 3 which is a two-branch version of Fig 2B in which we identify the activated state E*S of E*S′ with final products E + P or E + P′, referring to incorporation of the correct or incorrect nucleotide, respectively. Alternatively, Scheme 1 and Fig 3 can be though of as a two-branch version of Fig 2C but with a nonexponentially distributed *τ*.

The performance of the KPR process in this setting is quantified by the probability $P(t_{\text{p}}<t_{{\text{p}}^{\prime }})$ that the FPT *t*_p_ to the correct product state (E + P) is less than the FPT *t*_p′_ to the wrong product (E + P′).

Assuming that *τ* is a fixed value (a *δ*-function distribution) and using a standard approach of conditioning on the next state, as detailed in Appendix A2 of S1 Text and [28], we find

$$\begin{array}{cc} P(t_{\text{p}}<t_{{\text{p}}^{\prime }}) & =\frac{k_{1}}{k_{1}+k_{1}^{\prime }e^{(k_{-1}-k_{-1}^{\prime })\tau }}=\frac{k_{1}e^{-k_{-1}\tau }}{k_{1}e^{-k_{-1}\tau }+k_{1}^{\prime }e^{-k_{-1}^{\prime }\tau }}. \end{array}$$ (2)

The probability $P(t_{\text{p}}<t_{{\text{p}}^{\prime }})$ represents the probability that the correct nucleotide is incorporated, *i.e.*, the *accuracy* of the DNA replication process.

A critical feature of KPR is the sensitivity of accuracies to the unbinding rates *k*_−1_ and $k_{-1}^{\prime }$. In the DNA replication setting, the accuracy $P(t_{\text{p}}<t_{{\text{p}}^{\prime }})$ has a similar form to the Hill function $\frac{x^{n}}{x^{n}+a^{n}}$, except that the monomials are replaced by exponentials. Since the exponential function changes more rapidly than the power function, the accuracy $P(t_{\text{p}}<t_{{\text{p}}^{\prime }})$ has a sharp sigmoidal dependence on the difference $k_{-1}-k_{-1}^{\prime }$, as shown by the black curve in S1 Fig. Using the same conditioning approach, we can also calculate (see Appendix A2 in S1 Text) the mean first passage time (MFPT) E[t] to either product starting from the free state E:

$$\begin{array}{c} \begin{array}{cc} E[t] & =\frac{\frac{k_{1}}{k_{-1}}(1-e^{-k_{-1}\tau })+\frac{k_{1}^{\prime }}{k_{-1}^{\prime }}(1-e^{-k_{-1}^{\prime }\tau })+1}{k_{1}e^{-k_{-1}\tau }+k_{1}^{\prime }e^{-k_{-1}^{\prime }\tau }}\\ \; & \approx \frac{e^{k_{-1}\tau }}{k_{1}}(\frac{k_{1}}{k_{-1}}+\frac{k_{1}^{\prime }}{k_{-1}^{\prime }}+1), \end{array} \end{array}$$ (3)

where the approximation holds if $k_{1}e^{-k_{-1}\tau }\gg k_{1}^{\prime }e^{-k_{-1}^{\prime }\tau }$, which is typically the case in DNA replication, as $P(t_{\text{p}}<t_{{\text{p}}^{\prime }})<1$.

### T cell receptor signaling context

Next, we consider proofreading in the TCR signaling context. T cells form contacts with an antigen presenting cells (APC) and scan the surface of the APC for the presence of a foreign antigen (or epitope) (S). The TCR on the T cell membrane can bind to the epitope on the APC membrane, initiating a signaling cascade that leads to T cell activation. The activated T cell can then produce signaling molecules that trigger the immune response. If the APC does not present an antigen that corresponds to the TCR, the T cell should not be activated within the contact time *T* and disengages from the APC. There are three features of TCR signaling that are distinct from the DNA replication process. First, the APC is likely not to carry a foreign antigen (the correct or “cognate” substrate (S)). Consequently, cognate and incorrect (“noncognate”) peptide-MHC complexes (pMHC), defined as S and S′, are not present at the same time during an encounter and do not compete with each other for TCR binding. Second, the APC and the T cell have a finite contact duration *T*. The recognition process can only occur within this time window. Lastly, the activated states produce identical products, *i.e.*, downstream signals, regardless of whether the substrate (epitope) is the correct one or not. Therefore, the cell needs a strategy to discriminate the correct (cognate) and incorrect (noncognate) substrates based on the number of products generated within the contact time *T*.

First, assume that *T* is fixed as in previous literature [10] (we will relax this assumption later on). *Within the time window T*, the reaction diagram is represented by Eq (4).

$$\begin{array}{c} \begin{array}{cc} \text{E}+\text{S} & \underset{k_{-1}}{\overset{k_{1}}{⇌}}\text{ES}\overset{1/\tau }{⇝}{\text{E}}^{*}\text{S}\overset{k_{\text{p}}}{\to }{\text{E}}^{*}\text{S}+\text{P},\\ {\text{E}}^{*}\text{S} & \overset{k_{-1}^{*}}{\to }\text{E}+\text{S}. \end{array} \end{array}$$ (4)

Typically, TCR recognition is fairly sensitive since a few correct substrates (or epitopes) S on the APC membrane are able to activate the T cell. In the following, we will assume that each APC contains only one type of epitope, S or S′, while there are multiple TCRs that can bind to the substrate. Mathematically, the roles of substrate and TCR are equivalent. Tracking the state of the single substrate gives a similar simple stochastic process as in the DNA replication setting. This assumption allows us to reduce the number of possible states in the corresponding stochastic process.

We first consider two possible discrimination strategies, the FPT-based strategy and the product-based strategy, to quantify the output of the TCR recognition process.

#### FPT-based scenario

In this strategy, reaching the activated state E*S within time *T* is interpreted as T cell activation. The output of this strategy can be represented using the FPT to E*S state (*t*_a_) as $X_{\text{a}}=1_{t_{\text{a}}<T}$. The state *X*_a_ = 1 denotes an activated T cell while *X*_a_ = 0 indicates no response by the T cell. In general, we can define $X_{\text{a}}\left(t\right)=1_{t_{\text{a}}<t}$, and visualize the output as a binary signal changing over time, as shown in Fig 1B. To justify the FPT strategy biologically, we note that upon reaching the activated state, cofactors such as CD4 and CD8 stabilize the TCR-pMHC interaction, significantly reducing the unbinding rate $k_{-1}^{*}$. Therefore, the activated complex can then steadily produce downstream signals (products) and trigger T cell response.

#### Product-based scenario

This strategy, which is implicitly analyzed in [11], estimates the pMHC-TCR “affinity” by counting the number of product or signaling molecules produced within a given time. The output of this strategy is the number of products P(*T*) at time *T*, which takes on values in N, as is shown in Fig 1C. Consequently, in this more graded strategy, whether a T cell is activated is not described by a single, specific criterion.

Different strategies of interpreting the output requires us to define performance metrics that can compare different strategies on a common mathematical footing. We now define the performance metric that can be used to compare our two discrimination scenarios.

#### Performance metrics

In the case of FPT-based discrimination, it is natural to formulate the recognition problem as a hypothesis testing problem. We denote the binary input *ξ* = 1 if S is present and *ξ* = 0 if S′ is present. The competing hypotheses are then *H*_0_: *ξ* = 0 and *H*_*a*_: *ξ* = 1. The cell accepts *H*_0_ if *X*_a_ = 0. There is a canonical definition of sensitivity and specificity, *i.e.*, the true positive probability (TPP) and true negative probability (TNP). Given a fixed duration *T*, varying the processing time *τ* gives a family of binary classifiers (solutions to the hypothesis testing problem) corresponding to the KPR process. The receiver-operating characteristic curve (ROC) and the area under the curve (AUC) can be used to evaluate the overall performance of this family of classifiers. For a single classifier, we define the accuracy A as an average of specificity and sensitivity:

$$\begin{array}{c} \text{sensitivity}\equiv P(X=1|\xi =1), \end{array}$$ (5a)

$$\begin{array}{c} \text{specificity}\equiv P(X=0|\xi =0), \end{array}$$ (5b)

$$\begin{array}{c} A\equiv \text{accuracy}=\frac{1}{2}\text{sensitivity}+\frac{1}{2}\text{specificity}. \end{array}$$ (5c)

However, such metric only applies to strategies with binary output, but not the product-based strategy, as the output P(*T*) is non-binary. Kirby and Zilman propose a Fisher linear discriminant metric (*η*_FLD_) based on signal-to-noise ratios [11]. However, *η*_FLD_ takes on values in (0, ∞) and does not directly quantify the fidelity of transmission from the input *ξ* to the output P(*T*).

In order to compare both strategies on a common footing and represent the fidelity of discrimination directly, we propose using the mutual information I between the input and output, and the associated channel capacity *C*. The mutual information between two random variables *ξ* and *X* can be defined as [29]

$$\begin{array}{c} I(\xi ;X)=S(\xi ⊗X)-S(\xi ,X), \end{array}$$ (6)

where *ξ* ⊗ *X* is the joint random variable of *ξ* and *X*, assuming that *ξ* and *X* are independent, while *S*(*ξ*, *X*) is the joint Shannon entropy of *ξ* and *X*. The mutual information I(ξ;X) relies on the input distribution of *ξ*. Hence, one can define the channel capacity *C* as the supremum of the mutual information over all possible input distributions of *ξ* which only depends on the conditional probability distribution of *X* given *ξ*,

$$\begin{array}{c} C\left(X\;|\;\xi \right)=\underset{\xi }{\text{sup}}\;I\left(\xi ;X\right). \end{array}$$ (7)

There are two advantages of using mutual information and channel capacity over the Fisher linear discriminant metric *η*_FLD_. First, the mutual information is defined for both binary variables, as in the case of the first-passage time problem, and continuous variables, as in the case of the product-based discrimination problem. Second, in the specific scenario of binary input variables (cognate and noncognate substrates), the mutual information always takes values between 0 and 1 when measured in bits (log 2). A mutual information of 0 means that the output distribution is independent of the input, thus information of the output does not inform anything about the input. In other words, the distributions of outputs corresponding to different inputs are identical. By contrast, a value of 1 bit means that the output distributions corresponding to two different but equally probable inputs do not overlap. Consequently, the channel capacity provides a natural way to compare the product-based discrimination problem with the FPT problem and to quantify how well the system can distinguish correct substrates from incorrect ones.

In the limit in which the accuracy A→1 with binary input *ξ* and output *X*, we note that the mutual information I is approximately A (measured in bits) when $P\left(\xi =0\right)=P\left(\xi =1\right)=\frac{1}{2}$:

$$\begin{array}{c} \begin{array}{c} I\left(X_{\text{a}};\xi \right)\approx A\left(\text{bits}\right). \end{array} \end{array}$$ (8)

In this high accuracy limit, the channel capacity *C* is also close to the accuracy.

#### Stochastic simulations

In cases where we need to rely on stochastic simulations of the KPR processes to evaluate performance metrics, we implement the Gillespie algorithm [30] in julia [31]. The Gillespie algorithm tracks the state transitions of a Markov chain with exponential waiting times. In order to simulate the KPR process with a deterministic waiting time *τ*, we explicitly track all waiting times at each step and the time elapsed, updating the state by the smallest waiting time. After the state update, we re-evaluate all the waiting times and the time elapsed. The implementation is available at github.com/hsianktin/KPR.

In order to obtain the mutual information and channel capacity, we record the FPTs during the simulation, as well as the number of products produced by each simulation trajectory after a given time *T*. We simulated 10^4^ trajectories and use the empirical distributions of the FPTs and the number of products as surrogates for the true distributions. This allows us to compute the conditional probability distribution of output *X* given input *ξ*. Then, mutual information is computed using Eq (6). The channel capacity is obtained by a numerical optimization procedure with respect to the probability of *ξ* = 1, P(ξ=1) in the interval (0, 1).

The exact parameters used in each simulation will be specified in the corresponding figures. In general, we set *k*_−1_ = 1, $k_{-1}^{\prime }=2$, indicating that the unbinding rate of an incorrect substrate is only twice as fast as that of a correct substrate.

### Notation

We summarize the mathematical symbols and notation used in this study in Table 1. To distinguish similar symbols used in different contexts, we use context-specific subscripts. For example, the subscript a denotes the quantity relevant for FPTs to the activated state, while the subscript p refers to quantities relevant for FPTs to the product state and subscript th denotes a threshold-based FPT strategy. We also use superscript ^o^ to denote the optimal value of a quantity, *e.g.*, $A^{\text{o}}$ denotes the maximal accuracy and *τ*^o^ denotes the optimal processing time that achieves the maximal accuracy. Additionally, $\hat{x}$ denotes an estimate of the quantity *x*.

**Table 1:** Notation and mathematical symbols.

Symbol	Description
*ξ*	binary input variable, correct (*ξ* = 1) or incorrect (*ξ* = 0) substrate
*X*	binary output variable; activated (*X* = 1) or non-activated (*X* = 0)
*T*	contact duration between T cell and APC
*k*_1_/*k*_−1_	binding/unbinding rates of correct substrate to enzyme
$k_{1}^{\prime }/k_{-1}^{\prime }$	binding/unbinding rates of incorrect substrate
*k*_f_	activation rate of the multistep KPR process
*k*_p_	production rate of product
*k*_off_	unbinding rate of the intermediate complex (assumed to be *k*_−1_)
*m*	number of proofreading steps in the multistep KPR process
*τ*	processing time of the KPR process
*t*_a_	first-passage time to the activated state
*N*	number of binding-unbinding events in the KPR process
A	accuracy of the KPR process
I	mutual information between input and output
*C*	channel capacity of the KPR process

Here, the KPR process refers to the kinetic proofreading process with a deterministic processing time *τ*. The multistep KPR process refers to the original KPR process with multiple proofreading steps.

## Results

Application of our approach to the DNA replication scenario is straightforward and characterized by the accuracy in Eq (2) and the MFPT in Eq (3). In particular, we show that our approach exhibits the expected sensitivity of the accuracy to the unbinding rates *k*_−1_ and $k_{-1}^{\prime }$, as shown in S1 Fig. This sensitivity is a key feature of KPR in DNA replication. In this section, we focus on analysis of the TCR signaling scenario using our deterministic waiting time approach, with an emphasis on the comparison between the FPT-based and product-based strategies.

### Long processing time improves maximal accuracy in FPT-based strategy

For convenience, in FPT-based scenarios, we assume parameters $k_{1}=k_{1}^{\prime }<k_{-1}<k_{-1}^{\prime }$, *T* ≫ 1/*k*_1_ and let *N* = *Tk*_1_, which allows us to treat the recognition process as *N* cycles of a one-shot process. For each shot, an initial complex ES either unbinds with a probability of $1-e^{-k_{-1}^{\prime }\tau }$ or $1-e^{-k_{-1}\tau }$, or activates. Then, *N* independent cycles of the process yields the geometric failure (non-activation) probability (1−*e*^−*k*_−1_*τ*^)^*N*^ and success (activation) probability ${\left(1-e^{-k_{-1}\tau }\right)}^{N}$ when the substrate is correct. Similarly, we have the non-activation probability ${\left(1-e^{-k_{-1}^{\prime }\tau }\right)}^{N}$ and activation probability $1-{\left(1-e^{-k_{-1}^{\prime }\tau }\right)}^{N}$ when the substrate is incorrect. The specificity, *i.e.*, true negative probability, is given by ${\left(1-e^{-k_{-1}^{\prime }\tau }\right)}^{N}$, the non-activation probability when the substrate is incorrect. The sensitivity, *i.e.*, the true positive probability, is given by $1-{\left(1-e^{-k_{-1}\tau }\right)}^{N}$, the activation probability when the substrate is correct.

In the one-shot process (*N* = 1), the sensitivity and specificity are controlled by the processing time *τ*, with a larger *τ* leading to a higher specificity and a lower sensitivity, as illustrated in panel A in S2 Fig. The trade-off between sensitivity and specificity can be summarized by the area under the ROC curve calculated to be $\text{AUC}=k_{-1}^{\prime }/\left(k_{-1}+k_{-1}^{\prime }\right)$ using Eq (9).

$$\begin{array}{c} \text{sensitivity}=e^{-k_{-1}\tau }, \end{array}$$ (9a)

$$\begin{array}{c} \text{specificity}=1-e^{-k_{-1}^{\prime }\tau }, \end{array}$$ (9b)

$$\begin{array}{c} \text{AUC}=\int_{0}^{1}\text{sensitivity}\;\text{d}\left(\text{specificity}\right)=\frac{k_{-1}^{\prime }}{k_{-1}+k_{-1}^{\prime }}. \end{array}$$ (9c)

The AUC is not sensitive to the unbinding rates *k*_−1_ and $k_{-1}^{\prime }$, having a “Hill coefficient” of 1. In the case where $k_{-1}^{\prime }/k_{-1}=2$, the AUC is only 0.67, indicating a failure to accurately discriminate between the correct and incorrect substrates. This inability of one-shot process can be overcome by increasing the number of cycles *N* in the process, illustrated by plot B in S2 Fig.

In a multi-shot process, the sensitivity and specificity are explicitly given by Eqs (10a) and (10b) according to the prior discussion. However, no simple closed-form expression of the AUC is available. Instead, we consider the accuracy A defined by Eq (5), where we assume equal prior probabilities of the correct and incorrect substrates.

$$\begin{array}{c} \text{sensitivity}=1-{\left(1-e^{-k_{-1}\tau }\right)}^{N} \end{array}$$ (10a)

$$\begin{array}{c} \text{specificity}={\left(1-e^{-k_{-1}^{\prime }\tau }\right)}^{N} \end{array}$$ (10b)

$$\begin{array}{c} A\left(\tau ,N\right)=\frac{1}{2}-\frac{{\left(1-e^{-k_{-1}\tau }\right)}^{N}}{2}+\frac{{\left(1-e^{-k_{-1}^{\prime }\tau }\right)}^{N}}{2} \end{array}$$ (10c)

**Fig 4:**
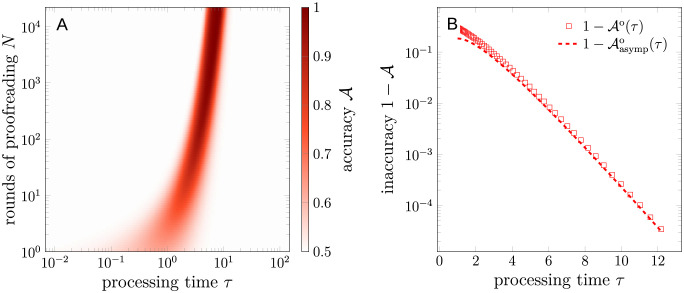
Statistics of the FPT-based strategy in the TCR recognition scenario. (A) Accuracy A as a function of processing time *τ* and contact duration *T*, evaluated using Eq (10). (B) The maximal accuracy $A^{\text{o}}$ (squares) as a function of processing time *τ*, evaluated using Eq (12). The asymptotic behavior of $A^{\text{o}}$ in the *τ* → ∞ limit, evaluated using Eq (13), is shown by the dashed curve. In (A,B), we set $k_{1}=k_{1}^{\prime }=0.1$, *k*_−1_ = 1, and $k_{-1}^{\prime }=2$.

The accuracy depends on both the number of binding events *N* (hence *T*) and processing time *τ*, as illustrated in Fig 4A. For fixed contact duration *T*, the accuracy first increases with *τ*, then decreases. In the long processing time limit *τ* → ∞, the T cell does not respond to any signal, corresponding to $A=\frac{1}{2}$.

For fixed processing time *τ*, there is a maximal accuracy $A^{\text{o}}\left(\tau \right)={\text{sup}}_{N}\;A\left(\tau ,N\right)$ and a corresponding $N^{\text{o}}\left(\tau \right)$ such that $A\left(\tau ,N^{\text{o}}\right)=A^{\text{o}}\left(\tau \right)$. A straightforward calculation yields

$$\begin{array}{c} \begin{array}{c} N^{\text{o}}\approx \frac{\left(k_{-1}^{\prime }-k_{-1}\right)\tau e^{k_{-1}\tau }}{1-e^{-(k_{-1}^{\prime }-k_{-1})\tau }},\;k_{-1}^{\prime }>k_{-1} \end{array} \end{array}$$ (11)

and

$$\begin{array}{c} \begin{array}{cc} A^{\text{o}}\left(\tau \right)\approx \frac{1}{2}+ & \frac{1}{2}{\left(1-e^{-k_{-1}^{\prime }\tau }\right)}^{\frac{(k_{-1}^{\prime }-k_{-1})\tau }{e^{-k_{\text{-1}}\tau }-e^{-k_{-1}^{\prime }\tau }}}(1-e^{-(k_{-1}^{\prime }-k_{-1})\tau }),\;k_{-1}^{\prime }>k_{-1}. \end{array} \end{array}$$ (12)

The asymptotic behavior of $A^{\text{o}}$ in the *τ* → ∞ limit is

$$\begin{array}{c} 1-A_{\text{asymp}}^{\text{o}}\left(\tau \right)\approx \frac{1}{2}(k_{-1}^{\prime }-k_{-1})\tau e^{-(k_{-1}^{\prime }-k_{-1})\tau },\;k_{-1}^{\prime }>k_{-1}. \end{array}$$ (13)

As indicated in Eq (13) and Fig 4, for the TCR recognition scenario, the *inaccuracy* or error probability scales with $e^{-(k_{-1}^{\prime }-k_{-1})\tau }$. The improvement in the accuracy comes at a cost of the increased total times spent in the proofreading process. The optimal contact duration *T*^o^ required for a specific *τ* is given by *N*^o^/*k*_1_, with *N*^o^ given by Eq (12). There is a common $e^{k_{-1}\tau }$ factor in both the inaccuracy and the optimal contact duration, indicating a trade-off between the accuracy and the total time *τ* spent in the proofreading process [see Fig 4B]. This observation is consistent with the accuracy-speed trade-off emphasized by Xiao and Galstyan [21]. The trade-off arises from an inability to perfectly discriminate between cognate and noncognate ligands in a one-shot process, which is constrained by the AUC of $k_{-1}^{\prime }/\left(k_{-1}+k_{-1}^{\prime }\right)$. Under a fixed contact time *T*, maximization of the accuracy corresponds to maximizing the average of the sensitivity and specificity by adjusting the processing time *τ*. The sensitivity and specificity under the optimal processing time should be balanced, as pushing one to the extreme will reduce the other to zero. For example, specificity in Eq (10b) approaches 1 when *τ* → ∞, while sensitivity in Eq (10a) approaches 0 in the same limit. Thus, by Eq (10c), the accuracy A(τ→∞,N)→1/2. Similarly, A(τ→0,N)→1/2.

### Channel capacity and Fisher linear discriminant agree qualitatively

We first show that the channel capacity metric and the Fisher linear discriminant metric show qualitatively similar behavior. To obtain statistically accurate results, we simulate the TCR recognition process using the Gillespie algorithm and evaluate the channel capacity and the Fisher linear discriminant metric from 10^4^ realizations. The results shown in S3 and S4 Figs indicate that both quantities increase with respect to cell-cell contact time *T* and are maximal at waiting time *τ* ∼ 0.6/*k*_−1_, regardless of *T*. We now establish the channel capacity as an appropriate metric for comparing different discrimination strategies.

### Invariant optimal processing time of product-based strategy for different contact times

**Fig 5:**
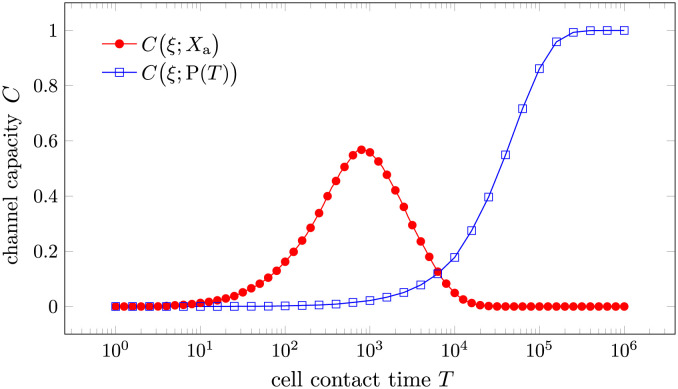
The channel capacity as a function of cell-cell contact time *T* for first-passage-time-based (FPT-based) signaling and product-based signaling. The channel capacity is evaluated between the input *ξ* indicating correct (1) or incorrect (0) substrate and the output *X*_a_ or P(*T*). We assumed $k_{1}=k_{1}^{\prime }=0.1$, $k_{-1}=k_{-1}^{*}=1$, $k_{-1}^{\prime }={\left[k_{-1}^{\prime }\right]}^{*}=2$, *τ* = 3, and $k_{\text{p}}=0.01$ for a slow product formation rate.

We now compare the channel capacity of FPT-based discrimination to that associated with product-based discrimination. In Fig 5 we plot the channel capacity between the input *ξ* and the outputs $X_{\text{a}}=1_{t_{\text{a}}\leq T}$ (in the FPT-based discrimination) and P(*T*) (in the product-based discrimination) as a function of cell contact time *T* when *τ* = 3 is fixed.

**Fig 6:**
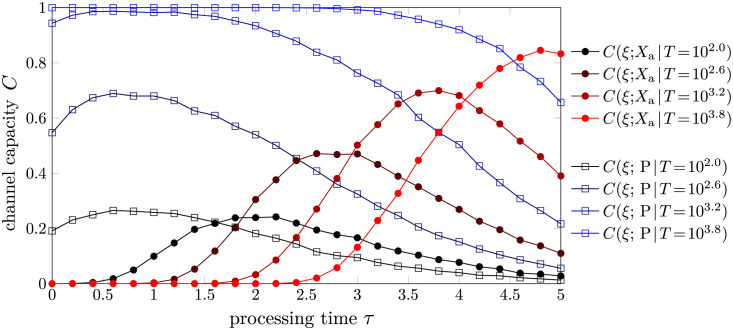
The channel capacities in product-based (blue squares) and first activation time-based (red dots) discrimination as a function of processing time *τ* for various cell contact times *T*. We evaluate the channel capacity using stochastic simulations (Gillespie algorithm) of the model in Eq (4) with parameters $k_{1}=k_{1}^{\prime }=0.1$, $k_{-1}=k_{-1}^{*}=1$, $k_{-1}^{\prime }={\left[k_{-1}^{\prime }\right]}^{*}=2$, and *k*_p_ = 1. The production rate was set higher for easier simulation of the product concentration.

In Fig 6, we plot the channel capacity as a function of processing time *τ* for various contact times *T*. As in Fig 5, we channel capacities associated with both FPT-based and product-based discrimination. The channel capacity of the product-based strategy increases monotonically with respect to cell contact time *T* while exhibiting a peak at $\tau_{\text{P}}^{\text{o}}∼0.6/k_{-1}$, as is shown previously. By contrast, the channel capacity under FPT-based discrimination has an optimal processing time $\tau_{\text{a}}^{\text{o}}$ that increases with respect to cell contact time *T*. The optimal contact time $T_{\text{a}}^{\text{o}}$ also increases with respect to the processing time *τ*. The channel capacity-optimizing contact times $T_{\text{a}}^{\text{o}},T_{\text{P}}^{\text{o}}$, and processing times $\tau_{\text{P}}^{\text{o}},\tau_{\text{a}}^{\text{o}}$ shown in panels A and B of S5 Fig.

There are two noteworthy observations from Fig 6. First, in the product-based scenario, the channel capacity for *τ* = 0 (no kinetic proofreading limit) does not differ significantly from the optimal channel capacity at $\tau_{\text{P}}^{\text{o}}$. This observation together with the *T*-independent optimal processing time $\tau_{\text{P}}^{\text{o}}$ reflects the conclusion by Kirby and Zilman that KPR is ineffective due to noise [11]. Second, under the same total contact duration *T*, the maximal channel capacity of the product-based strategy is higher than that of the FPT-based strategy provided that the production rate *k*_p_ is sufficiently large. These two observations suggest that the product-based discrimination is a superior strategy compared to the FPT-based one. In order to provide mechanistic insight into the difference between these two strategies, we now introduce a method to analytically characterize the product-based strategy.

### Decomposition of the product-based strategy

**Fig 7:**
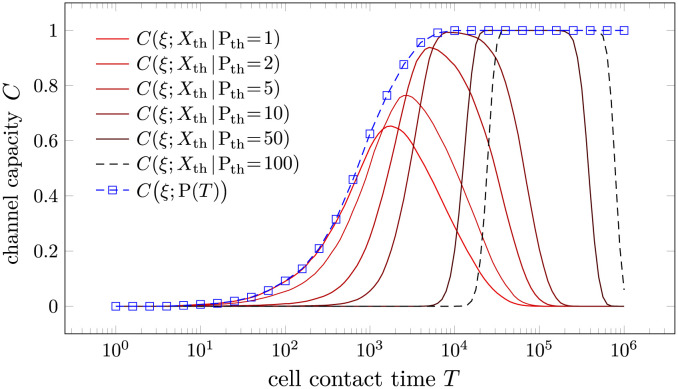
The channel capacity between the input *ξ* and the output P(*T*) or *X*_th_ as a function of cell-cell contact time *T*. Here, $k_{1}=k_{1}^{\prime }=0.1$, $k_{-1}=k_{-1}^{*}=1$, $k_{-1}^{\prime }={\left[k_{-1}^{\prime }\right]}^{*}=2$, *τ* = 3, and *k*_p_ = 1.

**Fig 8:**
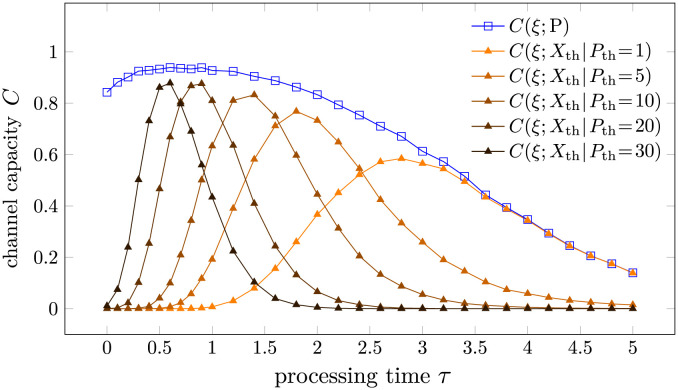
The channel capacity between the input *ξ* and the output *X*_a_, P(*T*) or *X*_th_ as a function of processing time *τ*. We assumed $k_{1}=k_{1}^{\prime }=0.1$, $k_{-1}=k_{-1}^{*}=1$, $k_{-1}^{\prime }={\left[k_{-1}^{\prime }\right]}^{*}=2$, *T* = 1000, and *k*_p_ = 1. 10,000 independent Gillespie simulations are conducted for each *τ*.

We propose decomposing the product-based strategy into a series of first-passage-time-based strategies using the FPT of the number of products P to different thresholds P_th_. Let *t*_*k*_ represent the first time the product P(*t*) exceeds the threshold *k*. X_th_ = 1 (triggering immune response) if $t_{{\text{P}}_{\text{th}}}\leq T$ and *X*_th_ = 0 otherwise. Being a FPT-based strategy, *C*(*ξ*; *X*_th_) has a similar dependence on *T* and *τ* to that of *C*(*ξ*; *X*_a_), the channel capacity in FPT-discrimination we discussed earlier. In order to illustrate this point, we perform Gillespie simulations of the model in Eq (4) with the same parameters as in Fig 6. The results are shown in Figs 7 and 8.

Since *X*_th_ is derived from P(*T*), *C*(*ξ*; P(*T*)) serves as an upper bound of *C*(*ξ*; *X*_th_) for various thresholds *P*_th_, as is illustrated in Figs 7 and 8. No single threshold can reach the information upper bound *C*(*ξ*; P(*T*)) at any *T*. A small threshold P_th_ can approach *C*(*ξ*; P(*T*)) for small *T* and large *τ*, while a large threshold P_th_ can approach the information upper bound *C*(*ξ*; P(*T*)) for large *T* and small *τ*. We thus introduce the approximation

$$\begin{array}{c} \hat{C}\left(\xi ;\text{P}\left(T\right)\right)≔\underset{{\text{P}}_{\text{th}}}{\text{max}}\;C\left(\xi ;X_{\text{th}}\right)\approx C\left(\xi ;\text{P}\left(T\right)\right) \end{array}$$ (14)

for *C*(*ξ*; P(*T*)).

### Mathematical analysis of the effects of *τ* in the product-counting strategy

Having established the approximation to the channel capacity of the product-based strategy in Eq (14), we further analyze its dependence on the processing time *τ* under a fixed cell-cell contact time *T*. We use the equivalence between channel capacity and accuracy in the high accuracy limit, as shown in Eq (8) to evaluate *C*(*ξ*; P(*T*)). We first introduce a Gaussian distribution approximation to the original distribution of P(*T*) with matched mean and variance

$$\begin{array}{c} E\left[\text{P}\left(T\right)\;|\;\xi =1\right]\approx \text{var}\left[\text{P}\left(T\right)\;|\;\xi =1\right]\approx k_{\text{p}}TKe^{-k_{-1}\tau },\;\;\;K\equiv \frac{k_{1}}{k_{1}+k_{-1}}. \end{array}$$ (15)

The *ξ* = 0 case is similar. The above steady-state approximation is justified in Appendix A1 of S1 Text.

Generating *P*_th_ in time $t_{{\text{P}}_{\text{th}}}<T$ is equivalent to P(*T*) ≥ P_th_. Thus, by approximating the distribution of P(*T*) by a normal distribution with mean E[P(T)] and variance var[P(*T*)], we can estimate the conditional probabilities of $X_{\text{th}}=1_{\text{P}\left(T\right)>{\text{P}}_{\text{th}}}$ given *ξ* by the integral

$$\begin{array}{cc} \text{sensitivity} & =P\left(X_{\text{th}}\;=1\;|\;\xi =1\right)\approx \int_{\frac{{\text{P}}_{\text{th}}-k_{\text{p}}TKe^{-k_{-1}\tau }}{\sqrt{k_{\text{p}}TKe^{-k_{-1}\tau }}}}^{\infty }\;e^{-x^{2}/2}\frac{\text{d}x}{\sqrt{2\pi }},\\ \text{specificity} & =P\left(X_{\text{th}}\;=0\;|\;\xi =0\right)\approx \int_{-\infty }^{\frac{{\text{P}}_{\text{th}}-k_{\text{p}}TK^{\prime }e^{-k_{-1}^{\prime }\tau }}{\sqrt{k_{\text{p}}TK^{\prime }e^{-k_{-1}^{\prime }\tau }}}}\;e^{-x^{2}/2}\frac{\text{d}x}{\sqrt{2\pi }}, \end{array}$$ (16)

where $K^{\prime }\equiv k_{1}^{\prime }/\left(k_{1}^{\prime }+k_{-1}^{\prime }\right)$. Under the assumption that $E\left[\text{P}\left(T\right)\;|\;\xi =1\right]>{\text{P}}_{\text{th}}$ and $E\left[\text{P}\left(T\right)\;|\;\xi =0\right]<{\text{P}}_{\text{th}}$, we can rewrite Eq (16) as

$$\begin{array}{c} \begin{array}{cc} P(X_{\text{th}}\;=1\;|\;\xi =1) & \approx \frac{1}{2}+\frac{1}{2}\text{erf}(\frac{k_{\text{p}}TKe^{-k_{-1}\tau }-{\text{P}}_{\text{th}}}{\sqrt{2k_{\text{p}}TKe^{-k_{-1}\tau }}}),\\ P(X_{\text{th}}\;=0\;|\;\xi =0) & \approx \frac{1}{2}+\frac{1}{2}\text{erf}(\frac{{\text{P}}_{\text{th}}-k_{\text{p}}TK^{\prime }e^{-k_{-1}^{\prime }\tau }}{\sqrt{2k_{\text{p}}TK^{\prime }e^{-k_{-1}^{\prime }\tau }}}). \end{array} \end{array}$$ (17)

In the high accuracy limit, the mutual information *C*(*ξ*; *X*_th_) between binary uniform input and binary output is approximated by the accuracy A as in Eq (8). We can then obtain the optimal threshold ${\text{P}}_{\text{th}}^{\text{o}}$ that maximizes the accuracy $A_{\text{th}}=P\left(X_{\text{th}}=1\;|\;\xi =1\right)+P\left(X_{\text{th}}=0\;|\;\xi =0\right)$ for given *T* and *τ* to be

$$\begin{array}{c} {\text{P}}_{\text{th}}^{\text{o}}\approx k_{\text{p}}T\sqrt{Ke^{-k_{-1}\tau }K^{\prime }e^{-k_{-1}^{\prime }\tau }}\;[1+O(T^{-1})]. \end{array}$$ (18)

The exact optimal threshold ${\text{P}}_{\text{th}}^{\text{o}}$ can be analytically solved, but we keep the above asymptotic form for simplicity when *T* → ∞. The maximal accuracy $A_{\text{th}}\left(\tau ,{\text{P}}_{\text{th}}={\text{P}}_{\text{th}}^{\text{o}}\right)$ is then used to approximate the maximal channel capacity ${\text{max}}_{{\text{P}}_{\text{th}}}C\left(\xi ;X_{\text{th}}\;|\;{\text{P}}_{\text{th}}\right)$, which is an approximation of the channel capacity *C*(*ξ*; P(*T*)) of the product-based discrimination. Upon substituting the zero-th order term of Eqs (18) into (17), we find

$$\begin{array}{c} \begin{array}{cc} \hat{C}\left(\xi ;\text{P}\left(T\right)\;|\;\tau \right) & ≔A_{\text{th}}\left(\tau ,{\text{P}}_{\text{th}}={\text{P}}_{\text{th}}^{\text{o}}\right)\\ \; & \approx \frac{1}{2}+\frac{1}{2}\text{erf}(\sqrt{\frac{k_{\text{p}}TKe^{-k_{-1}\tau }}{2}}-\sqrt{\frac{k_{\text{p}}TK^{\prime }e^{-k_{-1}^{\prime }\tau }}{2}}),\;\;\;k_{-1}^{\prime }>k_{-1}. \end{array} \end{array}$$ (19)

**Fig 9:**
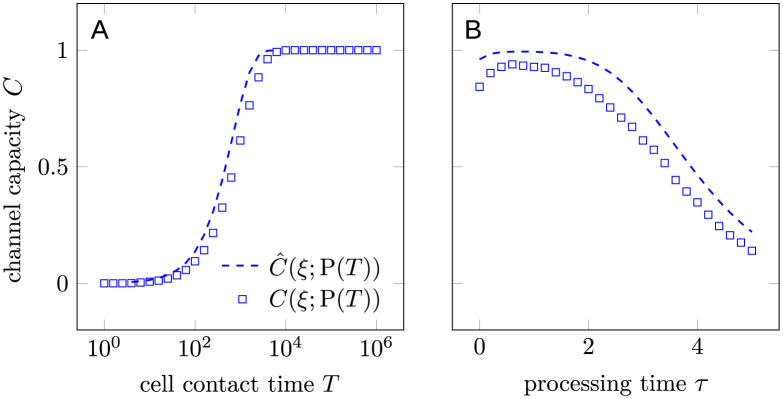
Comparison between the simulated channel capacity *C*(*ξ*; P(*T*)) and the corresponding estimate using Eq (19). (A) *C*(*ξ*; P(*T*)) as a function of cell-cell contact time *T*; (B) *C*(*ξ*; P(*T*)) as a function of processing time *τ*. Here, we took $k_{1}=k_{1}^{\prime }=0.1$, $k_{-1}=k_{-1}^{*}=1$, $k_{-1}^{\prime }={\left[k_{-1}^{\prime }\right]}^{*}=2$, and *k*_p_ = 1. *τ* = 3 in (A) and *T* = 1000 in (B).

In Fig 9, note that Eq (19) generally matches the simulation results well. Slightly higher values of Eq (19) arise from the Gaussian approximation used to map discrete output to continuous output.

The choice of optimal processing time ${\hat{\tau }}_{\text{P}}^{\text{o}}$ that maximizes the accuracy in Eq (19) is independent of *T* and is given by

$$\begin{array}{c} \begin{array}{cc} {\hat{\tau }}_{\text{P}}^{\text{o}} & \approx \frac{2}{k_{-1}^{\prime }-k_{-1}}\text{log}(\sqrt{\frac{K^{\prime }}{K}}\frac{k_{-1}^{\prime }}{k_{-1}}). \end{array} \end{array}$$ (20)

Note that the optimal processing time ${\hat{\tau }}_{\text{P}}^{\text{o}}$ is obtained by taking ${\text{P}}_{\text{th}}={\text{P}}_{\text{th}}^{\text{o}}\left(T\right)$ and is independent of the cell-cell contact time *T*. Under the parameter settings where $k_{1}=k_{1}^{\prime }=0.1$, *k*_−1_ = 1, and $k_{-1}^{\prime }=2$, we find that ${\hat{\tau }}_{\text{P}}^{\text{o}}$ is approximately 0.6/*k*_−1_, which is consistent with the optimal processing time $\tau_{\text{P}}^{\text{o}}$ obtained from the simulation results, as illustrated in S5 Fig.

As we have discussed and illustrated in Fig 6, the product-based discrimination strategy seems to be superior to the FPT-based strategy in the sense that the optimal processing time $\tau_{\text{P}}^{\text{o}}$ is independent of the cell-cell contact time *T* and the optimal channel capacity at a given *T* is higher than that of the FPT-based strategy. Through the decomposition of the product-based strategy by different thresholds, the first advantage of the product-based strategy, *i.e.*, invariant optimal *τ*, can be explained by the implicit assumption of the variability of the threshold P_th_. When *T* is small, a small threshold P_th_ is optimal, while a large threshold P_th_ is optimal when *T* is large. Changes in the threshold P_th_ allow the product-based strategy to adapt to different cell-cell contact times *T* without changing the processing time *τ*. By contrast, if the threshold P_th_ is fixed, then the dependence of the channel capacity on *T* and *τ* is similar to that of the FPT-based strategy, illustrated by the red curves in Figs 7 and 8. The importance of variability on the threshold P_th_ is even more pronounced when the total contact time *T* is random, as discussed in the next subsection.

### Dynamic thresholds under random cell-cell contact times

In the previous sections, we have assumed that the cell-cell contact time *T* is deterministic. In reality, the cell-cell contact time *T* is random and vary from cell to cell [32–34]. To evaluate the effects of a random cell-cell contact time, we consider a simple model where the cell-cell contact time *T* is uniformly distributed in the interval [0, *T*_max_], where *T*_max_ is the maximal cell-cell contact time.

**Fig 10:**
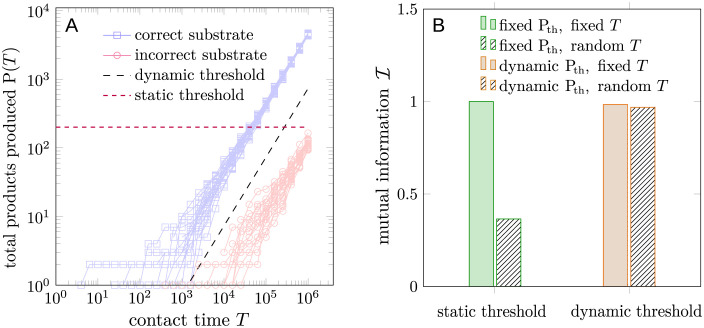
(A) Illustration of discrimination using a dynamic threshold ${\text{P}}_{\text{th}}^{\text{o}}$ as a function of time *T* since initial contact. The blue trajectories represent the number of products P with correct substrates. The red trajectories represent that of incorrect substrates. (B) A dynamic-threshold-based discrimination strategy maintains a high channel capacity when the total contact time *T* is uniformly distributed between 0 and *T*_max_. Filled bars represent the mutual information between input *ξ* and output *X*_th_ with a fixed contact time *T* and patterned bars represent the mutual information with a uniformly distributed contact time *T* between 0 and *T*_max_. The green bars indicate the maximal mutual information over all possible contact times *T* ≤ *T*_max_ and all possible static thresholds P_th_. The input *ξ* is assumed to be uniformly distributed on {0, 1}. We assumed $k_{1}=k_{1}^{\prime }=0.1$, $k_{-1}=k_{-1}^{*}=1$, $k_{-1}^{\prime }={\left[k_{-1}^{\prime }\right]}^{*}=2$, *τ* = 3, and *k*_p_ = 1. *T*_max_ is set to 10^6^. To filter out noisy transients, we additionally mandate that when the dynamic threshold ${\text{P}}_{\text{th}}^{\text{o}}\left(t\right)$ is smaller than 10 products, no response is initiated.

In the previous section, we conclude that the *T*-independent optimal processing time $\tau_{\text{P}}^{\text{o}}$ is a result of fixing the threshold P_th_ to ${\text{P}}_{\text{th}}^{\text{o}}\left(T\right)$ given by Eq (18). In the case of a random cell-cell contact time *T*, choosing a universally optimal threshold ${\text{P}}_{\text{th}}^{\text{o}}$ is difficult. We can, however, choose a dynamic threshold ${\text{P}}_{\text{th}}^{\text{o}}\left(t\right)$ that increases with the time *t* passed since the initial contact to maximize the channel capacity, where ${\text{P}}_{\text{th}}^{\text{o}}$ is still given by Eq (18), with *T* replaced by *t*. A comparison of a dynamic threshold ${\text{P}}_{\text{th}}^{\text{o}}\left(t\right)$ and a static threshold P_th_ is shown in Fig 10A. The numerical results of the maximal mutual information between the input and output *X*_th_ under different contact times *T* and different thresholds P_th_ are shown in Fig 10B. In the case of a fixed contact time *T*, the maximal mutual information of both static and dynamic thresholds is close to 1, indicating perfect discrimination. In the case of a uniformly distributed contact time *T* between 0 and *T*_max_, the maximal mutual information of the dynamic threshold is close to 1, while that of the static threshold is close to 0.4, indicating poor discrimination.

The experiments in Fig 10 suggest that the dynamic threshold ${\text{P}}_{\text{th}}^{\text{o}}\left(t\right)$ maintains a high channel capacity when the total contact time *T* is random.

### A nested single-binding KPR scheme

We have provided a mechanistic explanation for the different qualitative behavior between the channel capacities of first-passage-time-based and product-based strategies, in particular, why the optimal processing time $\tau_{\text{P}}^{\text{o}}$ is independent of the cell-cell contact time *T* in the product-based discrimination.

It is also of interest to understand the high channel capacity in the product-based discrimination problem that arises even in the *τ* → 0 limit, as shown by the blue curve of *C*(*ξ*; P) in Fig 8. Specifically, why does the product-based strategy carry a higher maximal channel capacity than the FPT-based strategy for a given *T* (as shown in Fig 6)? To provide simple mechanistic intuition, we can describe the production and accumulation of product by a sequence of events (*e.g.*, phosphorylation) within an effective KPR scheme:

$$\begin{array}{c} \begin{array}{cc} \text{APC}\;+\;\text{T} & \overset{{\tilde{k}}_{+1}}{\to }\;{\text{APC-T}}^{\left(0\right)}\;\overset{{\tilde{k}}_{\text{f}}}{\to }\;{\text{APC-T}}^{\left(1\right)}\;\;\to \cdots \to \;{\text{APC-T}}^{\left({\text{P}}_{\text{th}}\right)},\\ \;{\text{APC-T}}^{\left(k\right)} & \overset{1/T}{⇝}\text{APC}+\text{T},\;k\in \left\{0,1,\cdots ,{\text{P}}_{\text{th}}\right\}. \end{array} \end{array}$$ (21)

Here, “APC” represents the antigen-presenting cell and “T” denotes a T cell. The superscript (*i*) represents the number of products generated by the T cell. The T cell initiates responses when the number of products exceeds a threshold P_th_.

Although there are processing steps within each successive product state APC-T^(*i*)^, the overall nested scheme is structurally similar to the traditional KPR scheme shown in Fig 2A provided effective rates that incorporate the “internal” proofreading states are appropriately defined. The waiting times in state APC-T^(*i*)^ before transitioning to state APC-T^(*i*+1)^ can be approximated by effective, correct and incorrect “phosphorylation” rates ${\tilde{k}}_{\text{f}}\approx k_{\text{p}}Ke^{-k_{-1}\tau }$ and ${\tilde{k}}_{\text{f}}^{\prime }\approx k_{\text{p}}K^{\prime }e^{-k_{-1}^{\prime }\tau }$. This assignment of ${\tilde{k}}_{\text{f}},{\tilde{k}}_{\text{f}}^{\prime }$ are appropriate in the quasi-steady state limit within each group of KPR processing steps, leading to an approximately exponentially distributed waiting time $1/{\tilde{k}}_{\text{f}}$ between successive phosphorylations or synthesis of products. In addition, disassembly of each state occurs over a cell-cell contact time *T*, which is typically sharply (*not* exponentially) distributed since the APC-T cell unbinding process involves multiple steps and collective effects of adhesion membrane proteins. Finally, also note that the activation time of the TCR signaling process, given by the FPT $\tau_{{\text{P}}_{\text{th}}}$ to generate a number of products P*beyond* the threshold P_th_ is also approximately a constant when P_th_ is sufficiently large. Consequently, the competition between two processes with almost fixed timescales allows highly informative outputs compared to the traditional KPR scheme and Michaelis-Menten kinetics, as indicated in Eq (22), where ⇝ and ⇜ represent a (near) deterministic waiting time *τ*.

$$\begin{array}{ll} \text{Michaelis-Menten}: & \text{E}+\text{S}\leftarrow \text{ES}\to {\text{E}}^{*}\text{S},\\ \text{KPR}: & \text{E}+\text{S}\leftarrow \text{ES}\;⇝\;{\text{E}}^{*}\text{S},\\ \text{nested}\;\text{KPR}: & \text{E}+\text{S}⇜\text{ES}\;⇝\;{\text{E}}^{*}\text{S}. \end{array}$$ (22)

To further illustrate the role of a deterministic waiting time in the accuracy of the above process, we compare it with an exponential waiting time, *i.e.*, standard Michaelis-Menten (MM) kinetics in Appendix A3 of S1 Text. As for a comparison between the traditional KPR scheme and the nested KPR scheme, we note that in the limit of deterministic processing time, the nested KPR can achieve exact discrimination In other words, the product-based discrimination strategy introduces an additional layer of kinetic proofreading illustrated in Eq (21). Distinct from conventional kinetic proofreading, the unbinding and activation of this extra layer of kinetic proofreading are both nonexponential, carrying more information or “memory” of the previous events. This allows the product-based discrimination strategy to achieve higher channel capacity than the FPT-based strategy.

## Discussion and conclusions

In this paper, we have considered kinetic proofreading schemes for two classes of biological processes, DNA replication process and T cell signaling. Through a deterministic processing time assumption, we are able to analytically characterize the accuracy and speed of the kinetic proofreading process in both cases. This simplification captures the essential features of the kinetic proofreading process, *i.e.*, the accuracy of the process depends critically on the unbinding rates $\left(k_{-1},k_{-1}^{\prime }\right)$ of the correct and incorrect substrates, exemplified by Eqs (2), (13) and (19). In all cases, *k*_−1_ and $k_{-1}^{\prime }$ impacts the accuracy of the process exponentially through terms like $e^{-k_{-1}\tau }$ and $e^{-k_{-1}^{\prime }\tau }$. The curves plotting accuracy versus incorrect substrate degradation rate are shown in S1 Fig and exhibit switch-like behavior. Overall, our analysis indicates that how the output of a kinetic proofreading process is used to make a decision is crucial to the performance of KPR. We have shown that in the case of DNA replication, the specificity of the process is always exponentially dependent on the processing time *τ* and increasing specificity comes at the cost of replication speed.

In the case of TCR signaling, the trade-off between specificity and sensitivity shown in panel A of S2 Fig can be mitigated by increasing the number of allowed failed attempts *N* which is proportional to the cell-cell contact time *T*. The overall accuracy A of the signaling process is still exponentially dependent on the processing time *τ*, as illustrated by Eq (13). For longer processing time *τ*, the higher accuracy can only be achieved at the cost of the signaling speed by exponentially increasing the cell-cell contact time *T*, or equivalently, the number of allowed failed attempts *N* to the optimal value *N*^o^ indicated by Eq (11). Since the DNA replication process also compares one first-passage time to another, the trade-off between speed and accuracy shares very similar characteristics with the FPT strategy in the TCR signaling process, despite their different biological contexts (see S6 Fig).

A variant of the FPT-based strategy, extracting the extreme FPT in the presence of multiple substrates (antigens), has also been proposed [10]. The main goal in [10] is to determine sensitivity and specificity of TCR recognition of foreign antigens when also exposed to a sea of self-antigens. The self-antigens in [10] are assumed to bind much more weakly to the TCR than the foreign antigens. While we are primarily interested in discrimination between correct and incorrect substrates with similar binding affinities. This is a typical situation as T cells need to identify cancer cells that present mutated self antigens on their surface. In this case, the affinity of the corresponding TCR to the self antigen is expected to be similar to the affinity to the foreign antigen. The densities of the self and foreign antigens are also expected to be similarly low.

The amount of product produced can also be used to distinguish correct and incorrect substrates. To compare the performance of product-based discrimination to that of FPT-based discrimination, we introduced a channel capacity between the input *ξ* ∈ {0, 1} (incorrect, correct substrate) and the output *X*, which can be either $X_{\text{a}}=1_{t_{\text{a}}\leq T}$ or the number of products generated P(*T*). We established the connection between the channel capacity and the accuracy of the discrimination problem in the high accuracy limit in Eq (8). Thus, the dependence of the first-passage-time-based discrimination on the processing time and the cell-cell contact time both exhibit a single peak associated with the optimal processing time $\tau_{\text{a}}^{\text{o}}$ and the optimal cell-cell contact time $T_{\text{a}}^{\text{o}}$, respectively, as illustrated in Figs 5 and 6. The optimal values are obtained by maximizing the channel capacity, which implicitly assumes equal prior probabilities of correct and incorrect substrates. To maximize the channel capacity, the sensitivity and specificity can be neither too high nor too low, as pushing either to the extreme will reduce the channel capacity.

By contrast, the product-counting discrimination problem has a distinct monotonic increase of the channel capacity with respect to the cell-cell contact time *T* and the optimal processing time $\tau_{\text{P}}^{\text{o}}$ is independent of *T*. We find analytic approximations of the channel capacity by decomposing the product-based discrimination problem into a series of first-passage-time-based discrimination problems with different thresholds. This observation allows us to analytically approximate $\tau_{\text{P}}^{\text{o}}$ by Eq (20). The higher maximum channel capacity (under fixed *T*) of product-based discrimination compared to FPT-based discrimination shown in Fig 6 can be understood by an extra layer of single-shot kinetic proofreading, in which the disassembly and activation of the extra layers of kinetic proofreading are both nonexponential and carry more information or “memory” of previous events.

The channel capacity of the product-based discrimination strategy in our work shows qualitatively similar behavior as the Fisher linear discriminant (FLD) used by Kirby and Zilman [11], which is defined by the ratio of squared difference between the means of the products with correct and incorrect substrates to the sum of their variances. In this case, the channel capacity is peaked at a short processing time, then decreases with increasing processing time. This observation led Kirby and Zilman to conclude that more proofreading steps decreases the specificity of the TCR signaling process. However, Kirby and Zilman also investigated an alternative metric, the ratio of false positive rate to total activation rate, where the activation is defined by the number of products exceeding a threshold, *i.e.*, our FPT-based discrimination with a threshold P_th_. In our work, the ratio of false activation to total activation decreases with increasing processing time *τ*, while Kirby and Zilman’s result suggested that the ratio *increases* with more processing steps [see their Fig 4c in [11]], which is a contradiction. Recently, Xiao and Galstyan [21] suggested a mistake in Kirby and Zilman’s computation of the false activation/total activation ratio. Correcting this mistake, Xiao and Galstyan found that the ratio of false activation to total activation decreases with increasing processing time, consistent with our results. However, a mistake in the ratio of false positive rate to total activation does not negate the FLD, which remains a useful metric for the product-based discrimination strategy. As discussed in our work, the discrepancy between the FLD and the false activation rate to total activation rate ratio arises from different strategies of discriminating between correct and incorrect substrates.

Our TCR signaling model is a simplified scenario that assumes a deterministic cell-cell contact time *T* and a deterministic processing time *τ*. However, we conducted additional simulations that relaxed the deterministic processing time assumption by explicit modeling of the irreversible phosphorylation process in Appendix A4 of S1 Text. The results show qualitatively similar behavior as the simplified model. Additionally, we fixed the mean processing time and varied the number of processing steps *m* to explore the effect of the number of processing steps in KPR on the channel capacity. The results suggest that larger *m* increases the channel capacity of the FPT-based discrimination scheme, while the channel capacity of the product-based discrimination remains effectively unchanged, as shown in S7 Fig.

A random cell-cell contact time *T* may also impair the performance of the T cell in distinguishing correct ligands from incorrect ones. The dynamic threshold P_th_ may rescue this impaired performance by allowing the T cell to effectively adjust the threshold in time. This rescue is illustrated in Fig 10. However, implementing a dynamic threshold requires the T-cell to keep track of the duration since the initial contact with the APC to adjust the threshold P_th_ accordingly. The duration may be tracked by another series of similar mechanical or biochemical reactions on the membrane-membrane interface that are triggered by the membrane-membrane contact, as discussed in [35]. Experimentally, the presence of such a dynamic threshold can be detected by simultaneously measuring the number of products P(*T*), the total contact time *T* until full activation, and other markers indicating whether the T-cell is activated or not.

From a historical perspective, the Hopfield strategy was originally proposed to explain the high fidelity in DNA replication [1]. The strategy is similar to the product strategy but considers only the mean value of the product (production rate) under the steady state. The ratios of the mean production rates of the incorrect product to the correct product are used to estimate the probability of making an error in the DNA replication. In other words, Hopfield *implicitly* used the steady-state rate to compare the first passage times of the correct and incorrect substrates. The original treatments claim that the error probability can be at most the squared ratios of the dissociation rates of the correct to incorrect substrates, in steady state with one proofreading step. In our model for DNA proofreading, we need not reference the nonequilibrium steady state since the dynamics are described entirely by first passage times. This allows us to extract arbitrarily high accuracy by increasing the processing time *τ*, at an expense of the replication rate.

McKeithan [6] also described the TCR recognition process as a steady-state strategy. While the ratio of the mean production rates of the incorrect product to the correct product can be used as a performance metric, in the TCR recognition setting, it is not explicitly related to an error probability. McKeithan noted that larger ratios of mean production rates are associated with lower signal strength, but did not specify how their relationship determines T cell response. This was only recently addressed by Kirby and Zilman [11] through their signal-to-noise ratio. Our work provides a comprehensive set of metrics, including mutual information and accuracy, to assess how reaction rates determine signal strength and how different discrimination strategies contribute to antigen recognition. The setting and results presented by Kirby and Zilman represent a specific case of our theory, *i.e.*, the scenario of the product-based discrimination strategy.

The main observation that motivated our introduction of different strategies for discrimination is that TCR signaling is not an isolated process but is an integral component of a cellular reaction network. An analysis using ideas of information theory and transport could provide insight into a long-standing but often overlooked question: what type of information is transferred from the signaling process to the downstream pathways? Our work assumes that information is primarily transmitted as a binary signal that decides whether a T cell response is triggered, while Kirby and Zilman [11] assumed that the information is a continuous signal that reflects the strength of binding affinity between ligands and receptors. Further work is needed to identify the most appropriate information-theoretic framework for TCR signaling.

On a theoretical level, kinetic signaling schemes represent stochastic, biological implementations of the classical Maxwell’s demon [36], where the receptor is the demon that measures the affinity of the ligand to the receptor and sorts the ligands accordingly. The canonical Maxwell demon needs memory to measure both the position and time of a particle. In the case of a stochastic demon and the measurement of binding affinity, a memoryless exponential processing time seems to be able to provide a nonzero channel capacity to distinguish the correct and incorrect ligands. However, additional memory as provided by the nonequilibrium kinetic proofreading process (and non-exponentially distributed waiting time *τ*) enhances the channel capacity significantly. While previous literature has explored the idea of a Maxwell’s demon [37, 38] and energy-accuracy bounds in generalized KPR processes [39], the quantitative interplay between energy cost, memory, and information processing still lacks a suitable language and awaits future elucidation. In particular, we have not considered energy cost which may influence the preference in cells for different strategies of discriminating correct substrates from incorrect substrates.

## Supporting information

S1 Text Mathematical appendices. **Appendix A1**: Master equation for the stochastic KPR model. **Appendix A2**: Derivation of accuracy in the DNA replication scenario **Appendix A3**: Information transmitted by KPR and Michaelis-Menten schemes. **Appendix A4**: Multistep binding model.
(PDF)

S1 FigAccuracy as a function of $k_{-1}^{\prime }$.The variation in accuracy as the dissociation rate $k_{-1}^{\prime }$ of the incorrect product changes for both the DNA and TCR settings, with *k*_−1_ = 1. The accuracies are calculated using Eqs (2), (10) and (19) of the manuscript. The value of *N* in Eq (10) is determined by the optimal value given in Eq (11). We set $k_{1}=k_{1}^{\prime }=0.1$, *τ* = 5, $k_{\text{p}}=0.01$, and *T* = 10^6^.(EPS)

S2 FigPerformance characteristics of one-shot and multi-shot KPR.(A) The receiver operating characteristic (ROC) curve for the one-shot and multishot KPR model with *k*_−1_ = 1, $k_{-1}^{\prime }=2$, $k_{1}=k_{1}^{\prime }=0.1$. (B) The area under the curve (AUC) as a function of the number of KPR rounds *N*.(EPS)

S3 FigDependence of the *ξ*-P channel capacity *C* and the Fisher linear discriminant *η*_FLD_ on the processing time *τ* for different values of *T*.Both *C* (open squares) and *η*_FLD_ (filled circles) are evaluated via numerical simulations from 10^4^ trajectories. We assumed $k_{1}=k_{1}^{\prime }=0.1$, $k_{-1}=k_{-1}^{*}=1$, $k_{-1}^{\prime }={\left[k_{-1}^{\prime }\right]}^{*}=2$, *τ* = 3, and *k*_p_ = 1 for a fast product formation rate.(EPS)

S4 FigDependence of the *ξ*-P channel capacity *C* and the Fisher linear discriminant *η*_FLD_ on the cell-cell contact time *T*.Both the channel capacity (circles) and the Fisher linear discriminant (squares) are evaluated via numerical simulations from 10^4^ trajectories. We assumed $k_{1}=k_{1}^{\prime }=0.1$, $k_{-1}=k_{-1}^{*}=1$, $k_{-1}^{\prime }={\left[k_{-1}^{\prime }\right]}^{*}=2$, *τ* = 3, and *k*_p_ = 1 for a fast product formation rate. For *η*_FLD_, when *T* > 10^3^, their values are too high to be shown in the figure. We cropped the values of *η*_FLD_ to (0, 1) for better visualization.(EPS)

S5 FigRelationship between optimal contact and processing times.Symbols represent results from simulations over a total time horizon of 10^6^, while the dashed curves represent analytic approximations. (A) Dependence of optimal cell contact times $T_{\text{P}}^{\text{o}}$ and $T_{\text{a}}^{\text{o}}$ on processing time *τ* under product-based and FPT-based strategies. Since *C*(*ξ*; *P*) is nondecreasing with respect to *T*, the maximizing $T_{\text{P}}^{\text{o}}$ value is given as the upper limit of the simulation period. The approximation for $T_{\text{a}}^{\text{o}}$, ${\hat{T}}_{\text{a}}^{\text{o}}=N^{\text{o}}/K_{1}$, is given by Eq (11). (B) Dependence of optimal processing times $\tau_{\text{P}}^{\text{o}}$ and $\tau_{\text{a}}^{\text{o}}$ on cell contact time *T*. The analytic approximation ${\hat{\tau }}_{\text{P}}^{\text{o}}$ is given by Eq (20). The parameters used are the same as those in Fig 6.(EPS)

S6 FigMean total system lifetime as a function of inaccuracy $P(t_{\text{p}}\geq t_{{\text{p}}^{\prime }})$.The total duration (MFPT to any absorbing state) of the DNA replication process as a function of inaccuracy is evaluated using Eq (3) and is shown by the blue circles. The optimal contact duration *T*^o^ in the TCR recognition scenario as a function of inaccuracy $1-A^{\text{o}}$ (red squares) is evaluated using Eq (12) and the definition *N* = *k*_1_*T*. Here $k_{1}=k_{1}^{\prime }=0.1$, *k*_−1_ = 1, and $k_{-1}^{\prime }=2$.(EPS)

S7 FigDependence of the channel capacity between input *ξ* and different outputs on the number of proofreading steps *m*.Channel capacities are obtained from numerical simulations of the multistep binding model with 10^5^ trajectories for each set of parameters. We assumed $k_{1}=k_{1}^{\prime }=0.1$, $k_{-1}=k_{-1}^{*}=1$, $k_{-1}^{\prime }={\left[k_{-1}^{\prime }\right]}^{*}=2$, *τ* = 3, and *k*_p_ = 1. *T* is fixed at 1000. Dashed lines represent the channel capacities in the deterministic processing time (*m* → ∞) limit.(EPS)
