# Supplementary material for: Reliable ligand discrimination in stochastic multistep kinetic proofreading: First passage time vs. product counting strategies
Source: PLoS Comput Biol. 2024 Jun 10;20(6):e1012183. doi: 10.1371/journal.pcbi.1012183 (PMC11192422; doi:10.1371/journal.pcbi.1012183)
Supplement: S1 Text — Appendix A1: Master equation for the stochastic KPR model. Appendix A2: Derivation of accuracy in the DNA replication scenario Appendix A3: Information transmitted by KPR and Michaelis-Menten schemes. Appendix A4: Multistep binding model. (PDF) [file pcbi.1012183.s001.pdf]

S1 Text. Mathematical Appendices

A1 Master equation for the stochastic KPR model

Here, we consider the master equation associated with the multi-round proofreading model described in the TCR setting of the main text and derive numerical methods to evaluate the mean and variance of the number of products $\mathrm{P}(T)$ produced up to time T.

We use $\mathbb{P}(n, \mathrm{E}+\mathrm{S}; t)$ to denote the probability at time t that the system is in the $\mathrm{E}+\mathrm{S}$ state and that exactly n products have been produced. Similarly, we use $\mathbb{P}(n, \mathrm{ES}; t)$ and $\mathbb{P}(n, \mathrm{E^*S}; t)$ to denote the probability of n products produced and the system is in the ES and $\mathrm{E^*S}$ states, respectively. However, since our model involves a deterministic processing time τ, we introduce the age-density probability function $\rho(n, a; t)$, where a indicates the age of the complex since its formation. $\mathbb{P}(n, \mathrm{ES}; t)$ and $\mathbb{P}(n, \mathrm{E^*S}; t)$ are then given by

$$\mathbb{P}(n, \mathrm{ES}; t) = \int_0^\tau \rho(n, a; t)\mathrm{d}a, \quad \mathbb{P}(n, \mathrm{E^*S}; t) = \int_\tau^\infty \rho(n, a; t)\mathrm{d}a. \tag{A1}$$

The off rates k_{-1} and k_{-1}^* can be considered as a death rate of the age-structured complexes ES and $\mathrm{E^*S}$, while the on rate k_1 times the probability of the $\mathrm{E}+\mathrm{S}$ state is a birth rate of ES. Consequently, an age-structured master equation can be written as

$$\begin{aligned}
\frac{\mathrm{d}}{\mathrm{d}t}\mathbb{P}(n, \mathrm{E}+\mathrm{S}; t) &= -k_1\mathbb{P}(n, \mathrm{E}+\mathrm{S}; t) + k_{-1}\mathbb{P}(n, \mathrm{ES}; t) + k_{-1}^*\mathbb{P}\left(n, \mathrm{E^*S}; t\right), \\
\partial_t\rho(n, a; t) + \partial_a\rho(n, a; t) &= -k_{-1}\rho(n, a; t), \quad a \le \tau, \\
\rho(n, 0; t) &= k_1\mathbb{P}(n, \mathrm{E}+\mathrm{S}; t), \\
\partial_t\rho(n, a; t) + \partial_a\rho(n, a; t) &= -k_{-1}^*\rho(n, a; t) - k_\mathrm{p}\rho(n, a; t) + k_\mathrm{p}\rho\left(n-1, a; t\right), \quad a > \tau,
\end{aligned} \tag{A2}$$

where k_p is the production rate of P by state $\mathrm{E^*S}$ (which does not alter the $\mathrm{E^*S}$ state).

By integrating Eqs (A2) over age a and summing over n, we find the set of "zero-th" moment equations for the probabilities

$$\begin{aligned}
\frac{\mathrm{d}}{\mathrm{d}t}\mathbb{P}(\mathrm{E}+\mathrm{S}; t) &= -k_1\mathbb{P}(\mathrm{E}+\mathrm{S}; t) + k_{-1}\mathbb{P}(\mathrm{ES}; t) + k_{-1}^*\mathbb{P}\left(\mathrm{E^*S}; t\right), \\
\frac{\mathrm{d}}{\mathrm{d}t}\mathbb{P}(\mathrm{ES}; t) &= k_1\mathbb{P}(\mathrm{E}+\mathrm{S}; t) - k_1\mathbb{P}(\mathrm{E}+\mathrm{S}; t-\tau)e^{-k_{-1}\tau} - k_{-1}\mathbb{P}(\mathrm{ES}; t), \\
\frac{\mathrm{d}}{\mathrm{d}t}\mathbb{P}(\mathrm{E^*S}; t) &= k_1\mathbb{P}(\mathrm{E}+\mathrm{S}; t-\tau)e^{-k_{-1}\tau} - k_{-1}^*\mathbb{P}(\mathrm{E^*S}; t),
\end{aligned} \tag{A3}$$

where we have used the solution $\rho(n, \tau; t) = \rho(n, 0; t-\tau)e^{-k_{-1}\tau} = k_1\mathbb{P}(n, \mathrm{E}+\mathrm{S}; t-\tau)e^{-k_{-1}\tau}$ for the $a \le \tau$ equation in (A2). Eqs (A3) represent a set of linearly coupled delay differential equations which can be further simplified by summing over product numbers n. For example, marginalizing over product numbers, $\mathbb{P}(Y; t) \equiv \sum_{n=0}^\infty \mathbb{P}(n, Y; t)$ and applying Laplace transforms $\tilde{\mathbb{P}}(Y; s) \equiv \int_0^\infty \mathbb{P}(Y; t)e^{-st}\mathrm{d}t$, we find

$$\tilde{\mathbb{P}}(\mathrm{E^*S}; s) = \frac{k_1 e^{-(s+k_{-1})\tau}}{(s+k_{-1}^*)\left(s + k_1 - \frac{k_1 k_{-1}}{s+k_{-1}}\left(1 - e^{-(s+k_{-1})\tau}\right) - \frac{k_1 k_{-1}^*}{s+k_{-1}^*}e^{-(s+k_{-1})\tau}\right)}. \tag{A4}$$

For $t \gg 1/(k_1 + k_{-1})$, after which initial transients of the solution have decayed, we find the steady-state probability of being in the E^*S state as

$$\begin{aligned}\mathbb{P}(\mathrm{E^*S}; t \to \infty) &= \lim_{s \to 0^+} \left[s\tilde{\mathbb{P}}(\mathrm{E^*S}; s) \right] \\ &= \frac{\frac{k_1}{k_{-1}^*} e^{-k_{-1}\tau}}{\frac{k_1}{k_{-1}^*} e^{-k_{-1}\tau} + \frac{k_1}{k_{-1}} \left(1 - e^{-k_{-1}\tau}\right) + 1}.\end{aligned} \tag{A5}$$

Similarly, we can construct equations for higher moments $\langle n^\alpha(t) \,|\, Y \rangle = \sum_{n=0}^{\infty} n^\alpha \mathbb{P}(n, Y; t)$ and define expected product counts as

$$\begin{aligned}\mathbb{E}[\mathrm{P}^\alpha(t)] \equiv& \langle n^\alpha(t) \rangle \\ =& \sum_{n=0}^{\infty} n^\alpha \left[\mathbb{P}(n, \mathrm{E+S}; t) + \mathbb{P}(n, \mathrm{ES}; t) + \mathbb{P}(n, \mathrm{E^*S}; t) \right] \\ \equiv& \langle n^\alpha(t) \,|\, \mathrm{E+S} \rangle + \langle n^\alpha(t) \,|\, \mathrm{ES} \rangle + \langle n^\alpha(t) \,|\, \mathrm{E^*S} \rangle.\end{aligned} \tag{A6}$$

By taking such moments over Eqs (A3) and using the Laplace-transform, one can solve for $\langle \tilde{n}^\alpha(s) \,|\, Y \rangle$ and find that $\langle \tilde{n}(s) \rangle$ has double real-valued poles at $s_* = 0$ and near $s_* \sim -(k_1 + k_{-1})$ (assuming $k_{-1}^* \sim k_{-1}$), leading to dynamics of the form $\langle n(t) \rangle \sim t e^{s_* t}$. Thus, after transients of duration $\sim 1/(k_1 + k_{-1})$, the expected cumulative product increases linearly with time.

It is also informative to consider the direct time evolution of the expected values of products. Taking time derivatives of Eq (A6) and using Eqs (A3), we find

$$\frac{\mathrm{d}}{\mathrm{d}t} \langle n(t) \rangle = k_{\mathrm{p}} \mathbb{P}(\mathrm{E^*S}; t), \tag{A7a}$$

$$\frac{\mathrm{d}}{\mathrm{d}t} \left\langle n^2(t) \right\rangle = k_{\mathrm{p}} \mathbb{P}\left(\mathrm{E^*S}; t\right) + 2k_{\mathrm{p}} \sum_{n=0}^{\infty} n \mathbb{P}\left(n, \mathrm{E^*S}; t\right) \tag{A7b}$$

where $\mathbb{P}(\mathrm{E^*S}; t) \equiv \sum_{n=0}^{\infty} \mathbb{P}(n, \mathrm{E^*S}; t)$ is the marginal probability of the E^*S state.

As suggested by the analyses of Eq (A4) and $\langle \tilde{n}^\alpha(s) \,|\, Y \rangle$ and the transients in $\langle n^\alpha(t) \,|\, Y \rangle$, our reaction exhibits a timescale separation. When $k_{\mathrm{p}} \ll k_{-1}^*$, we expect the probability to cycle through the states $\mathrm{E+S} \to \mathrm{ES} \to \mathrm{E^*S} \to \mathrm{E+S}$ many times before a new product is generated. Thus, we can integrate Eq (A7a) using the steady-state approximation for $\mathbb{P}(\mathrm{E^*S}; t \to \infty)$ given in Eq (A5):

$$\langle n(t) \rangle \approx t\mathbb{P}(\mathrm{E^*S}; t \to \infty), \text{ as } t \to \infty. \tag{A8}$$

Additionally, separation of time scales allows us to approximate

$$\langle n(t) | Y \rangle \approx \langle n(t) \rangle \mathbb{P}(Y; t) = \mathbb{P}(\mathrm{E^*S}; t \to \infty) t \mathbb{P}(Y; t \to \infty). \tag{A9}$$

Then, Eq (A7b) can be expressed as

$$\frac{\mathrm{d}}{\mathrm{d}t} \langle n^2(t) \rangle = 2k_{\mathrm{p}} \langle n(t) | \mathrm{E^*S} \rangle + k_{\mathrm{p}} \mathbb{P}(\mathrm{E^*S}; t) \approx 2k_{\mathrm{p}} \left[\mathbb{P}(\mathrm{E^*S}; t \to \infty) \right]^2 t + k_{\mathrm{p}} \mathbb{P}(\mathrm{E^*S}; t). \tag{A10}$$

Since $\langle n^2(t=0) \rangle = 0$, we can integrate the above equation to find

$$\langle n^2(t) \rangle = k_{\mathrm{p}} \mathbb{P}(\mathrm{E^*S}; t) t \Big[1 + \mathbb{P}(\mathrm{E^*S}; t \to \infty) t \Big] \tag{A11}$$

In other words, the variance of P at time t is given by

$$\mathrm{Var}[\mathrm{P}(t)] = \mathbb{P}(\mathrm{E^*S}; t \to \infty) t = \mathbb{E}[\mathrm{P}(t)]. \tag{A12}$$

One can further verify that the distribution of $\mathrm{P}(t)$ should be Poisson-like in the small k_{p} limit.

A1.1 Limit of discrete processes

As we have discussed, our deterministic processing time τ can be considered as the infinite-number limit of discrete-step KPR processes with $k_\mathrm{f} = \frac{m}{\tau}$, $m \to \infty$. When $k_{-1} = k_{-1}^*$, from [11], the expressions for the mean and variance of the product P at time T were found to be

$$\begin{aligned} \langle n(t)\rangle_m =& k_\mathrm{p} t \left(\frac{k_\mathrm{f}}{k_\mathrm{f}+k_{-1}}\right)^m \frac{k_1}{k_1+k_{-1}}, \\ \sigma_m^2(t) =& \langle n(t)\rangle_m \left(1+2\frac{k_\mathrm{p}}{k_\mathrm{off}}\right) + \frac{2tk_\mathrm{p}^2\left(\frac{k_1}{k_{-1}}\right)^2\left(2+\frac{k_1}{k_{-1}}+\frac{k_\mathrm{off}}{k_\mathrm{f}}\left(2+m+\frac{k_1}{k_{-1}}+m\frac{k_1}{k_{-1}}\right)\right)}{\left(1+\frac{k_{-1}}{k_\mathrm{f}}\right)^{2m+1}\left(1+\frac{k_1}{k_{-1}}\right)^3 k_\mathrm{off}}. \end{aligned} \tag{A13}$$

Setting $k_\mathrm{f} = \frac{m}{\tau}$ and taking the $m \to \infty$ limit yields

$$\begin{aligned} \langle n(T)\rangle_m &\to \frac{k_\mathrm{p} T e^{-k_\mathrm{f}} k_1}{k_1+k_{-1}}, \\ \sigma_m^2(T) &\to \langle n(T)\rangle_m \left(1+\frac{2k_\mathrm{p}}{k_{-1}}+2\frac{\tau}{T}\langle n(T)\rangle_m\right), \end{aligned} \tag{A14}$$

which shows that when $\tau \ll T$ and $k_\mathrm{p}/k_{-1} \ll 1$, both the mean and variance approach $\langle n(T)\rangle_m$, recovering the results in Eq (A8) and Eq (A12).

A2 Derivation of accuracy in the DNA replication scenario

We denote $\mathbb{P}(t_\mathrm{p} < t_{\mathrm{p}'} \,|\, X)$ as the probability that the stopping times $t_\mathrm{p} < t_{\mathrm{p}'}$ starting from the state X, which can be either E, ES, etc. The probabilities to the absorbing states starting from different initial states are related by the one-step transition probabilities between the states. For a given state X, let Ω_X denote the set of states that can be reached from X in one step. Let $\mathbb{P}(Y \,|\, X)$ denote the one-step transition probability from X to Y, for $Y \in \Omega_X$. Then, the probabilities $\mathbb{P}(t_\mathrm{p} < t_{\mathrm{p}'} \,|\, X)$ satisfy the following equation:

$$\mathbb{P}(t_\mathrm{p} < t_{\mathrm{p}'} \,|\, X) = \sum_{Y \in \Omega_X} \mathbb{P}(Y \,|\, X)\mathbb{P}(t_\mathrm{p} < t_{\mathrm{p}'} \,|\, Y). \tag{A15}$$

This set of equations can be solved by imposing the boundary conditions $\mathbb{P}(t_\mathrm{p} < t_{\mathrm{p}'} \,|\, \mathrm{E}+\mathrm{P}) = 1$ and $\mathbb{P}(t_\mathrm{p} < t_{\mathrm{p}'} \,|\, \mathrm{E}+\mathrm{P}') = 0$, which hold because t_p is the first passage time to the state $\mathrm{E}+\mathrm{P}$ and $t_{\mathrm{p}'}$ is the first passage time to the state $\mathrm{E}+\mathrm{P}'$. In general, for Markovian transitions, the probability that state Y is first reached starting from state X is $\mathbb{P}(Y \,|\, X) = \frac{W_{Y,X}}{\sum_{Y' \in \Omega_X} W_{Y',X}}$ where $W_{Y,X}$ is the transition rate from X to Y and Y' denotes all states directly accessible from X. However, when both Markovian and deterministic-waiting-time processes compete with each other, the transition probabilities to each state need to be calculated explicitly. If a starting state X can transition to two different final states, with one transition being Markovian with rate k and waiting time τ_k and the other being deterministic with waiting time τ, the probability that the Markovian process occurs is $\mathbb{P}(\tau_k \le \tau) = \int_0^\tau k e^{-kt}\mathrm{d}t = 1 - e^{-k\tau}$. Using these considerations, we can systematically derive Eq (2) from

$$\mathbb{P}(t_\mathrm{p} < t_{\mathrm{p}'} \,|\, \mathrm{E}) = \frac{k_1}{k_1+k_1'}\mathbb{P}(t_\mathrm{p} < t_{\mathrm{p}'} \,|\, \mathrm{ES}) + \frac{k_1'}{k_1+k_1'}\mathbb{P}(t_\mathrm{p} < t_{\mathrm{p}'} \,|\, \mathrm{ES}') \tag{A16a}$$

$$\mathbb{P}(t_\mathrm{p} < t_{\mathrm{p}'} \,|\, \mathrm{ES}) = e^{-k_{-1}\tau} + \left(1 - e^{-k_{-1}\tau}\right)\mathbb{P}(t_\mathrm{p} < t_{\mathrm{p}'} \,|\, \mathrm{E}) \tag{A16b}$$

$$\mathbb{P}(t_\mathrm{p} < t_{\mathrm{p}'} \,|\, \mathrm{ES}') = (1 - e^{-k'_{-1}\tau})\mathbb{P}(t_\mathrm{p} < t_{\mathrm{p}'} \,|\, \mathrm{E}). \tag{A16c}$$

Substituting Eqs (A16b) and (A16c) into Eq (A16a), we obtain Eq (2).

Similarly, the derivation of the first passage time to either $\mathrm{E}+\mathrm{P}$ or $\mathrm{E}+\mathrm{P}'$ in Eq (3) can be obtained by solving the following set of equations

$$\mathbb{E}[t \mid \mathrm{E}] = \frac{1}{k_1+k_1'} + \frac{k_1}{k_1+k_1'}\mathbb{E}[t \mid \mathrm{ES}] + \frac{k_1'}{k_1+k_1'}\mathbb{E}[t \mid \mathrm{ES}'] \tag{A17a}$$

$$\mathbb{E}[t \mid \mathrm{ES}] = \frac{1-e^{-k_{-1}\tau}}{k_{-1}} + \left(1-e^{-k_{-1}\tau}\right)\mathbb{E}[t \mid \mathrm{E}] \tag{A17b}$$

$$\mathbb{E}[t \mid \mathrm{ES}'] = \frac{1-e^{-k'_{-1}\tau}}{k'_{-1}} + (1-e^{-k'_{-1}\tau})\mathbb{E}[t \mid \mathrm{E}]. \tag{A17c}$$

Here, $\mathbb{E}[t \mid X]$ denotes the expected first passage time to either $\mathrm{E}+\mathrm{P}$ or $\mathrm{E}+\mathrm{P}'$ starting from the state X. The boundary conditions are $\mathbb{E}[t \mid \mathrm{E}+\mathrm{P}]=0$ and $\mathbb{E}[t \mid \mathrm{E}+\mathrm{P}']=0$. The first "source" terms in each equation denote the expected time before leaving the current state X. Specifically, the expected time to leave the state E is the mean of the minimum of two exponential random variables. Since the minimum of two exponential random variables with rates k and k' is an exponential random variable with rate $k+k'$, the expected time in state E before leaving is $1/(k_1+k_1')$. The expected dwell times in states ES and ES$'$ are the expected minimum of an exponentially distributed random variable and the deterministic time τ. For example, the dwell time in state ES can be explicitly calculated as $\int_0^\tau s k_{-1} e^{-k_{-1}s}\mathrm{d}s + \int_\tau^\infty \tau k_{-1}e^{-k_{-1}s}\mathrm{d}s = \left(1-e^{-k_{-1}\tau}\right)/k_{-1}$.

A3 Information transmitted by KPR and Michaelis-Menten schemes

To illustrate why the simplified KPR scheme performs better than the Michaelis-Menten (MM) scheme, which are different only through a different choice of proofreading/processing time τ, consider the DNA replication scenario. We will provide a mathematical analysis and physical intuition from an information-theoretic perspective. To be specific, we consider the following two schemes:

$$\text{KPR: } \mathrm{E}+\mathrm{S} \leftrightarrow \mathrm{ES} \overset{1/\tau}{\rightsquigarrow} \mathrm{E}+\mathrm{P}, \tag{A18}$$

$$\text{MM: } \mathrm{E}+\mathrm{S} \leftrightarrow \mathrm{ES} \overset{k_\mathrm{f}}{\to} \mathrm{E}+\mathrm{P}. \tag{A19}$$

Mathematical analysis. In the scenario of DNA replication, note that Scheme (A18) yields an error probability of

$$P_{\mathrm{e,KPR}} = \frac{k_1' e^{(k_{-1}-k'_{-1})\tau}}{k_1 + k_1' e^{(k_{-1}-k'_{-1})\tau}} \to 0, \text{ as } \tau\to\infty. \tag{A20}$$

On the other hand, Scheme (A19) yields an error probability of

$$P_{\mathrm{e,MM}} = \frac{k_1' k_\mathrm{f}}{(k_1+k_1')(k'_{-1}+k_\mathrm{f}) - k_1 k_{-1}\left(\frac{k'_{-1}+k_\mathrm{f}}{k_{-1}+k_\mathrm{f}}\right) - k_1' k'_{-1}}. \tag{A21}$$

By investigating $\frac{\mathrm{d}P^{-1}_{\mathrm{e,MM}}}{\mathrm{d}k_\mathrm{f}}$, we find that $P_{\mathrm{e,MM}}$ is monotonically increasing with respect to k_f, with the maximum $P_{\mathrm{e,MM}}(k_\mathrm{f}\to\infty) = k'_{-1}/(k_1+k_1')$ and minimum $P_{\mathrm{e,MM}}(k_\mathrm{f}\to 0) = \frac{k_1'}{(k_1+k_1')+\frac{k_1}{k_{-1}}(k'_{-1}-k_{-1})}$.

To summarize, the error probability of Scheme (A18) converges to 0 at an exponentially fast rate, while the error probability of Scheme (A19) converges to a positive limit at a finite rate. In the no-proofreading limit, $k_\mathrm{f}=\infty$ and $\tau=0$, both schemes yield $k'_{-1}/(k_1+k_1')$, which depends only on the binding rate.

Physical intuition. Next, we provide an intuitive explanation of the above results from an information-theoretic perspective. To simplify the analysis, we consider the information yield of the two schemes *per binding-unbinding cycle*. We begin by introducing a timer a that records the time spent in the bound state (ES).

At time $t = 0$, we set the system in the bound state and set the timer a to 0. The unbinding waiting time τ_{-1} is exponential with rate k_{-1} or k'_{-1}, depending on the input $\xi = 1$ or $\xi = 0$. At the moment of unbinding, the timer $a = \tau_{-1}$ is read and used to determine whether the product is formed. In other words, this can be viewed as a two-step channel with the first step being the reading of the timer a and the second step being the determination of the product formation, which maps the timer value to the product formation.

The information yield of the first step is given by the mutual information between the input ξ and the timer (or age) a, in which we assume ξ is uniformly distributed. In the case of $k_{-1} = 1$ and $k'_{-1} = 2$, the mutual information is given by $\mathcal{I}(\xi; a) \approx 0.078$.

The second step involves a mapping from the timer value to the product formation. In the case of Scheme (A18), the mapping is deterministic with a threshold τ, given by $\mathbb{1}_{a>\tau}$. In the case of Scheme (A19), the mapping is stochastic with an additional exponential waiting time τ_f with rate k_f, given by $\mathbb{1}_{a>\tau_\mathrm{f}}$. We can expect that because of the additional layer of stochasticity, the information yield of the MM scheme is much lower than that of the KPR scheme, as confirmed by the numerical results in Fig A1. We also note that the information yield per binding-unbinding cycle is low. Thus, multiple

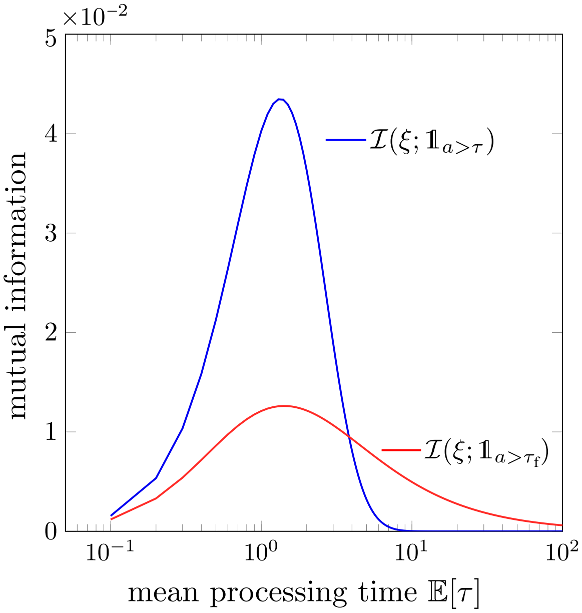

Fig A1. The mutual information between the input ξ and the final output for the KPR and Michaelis-Menten schemes ($\mathcal{I}(\xi; \mathbb{1}_{a>\tau})$, $\mathcal{I}(\xi; \mathbb{1}_{a>\tau_\mathrm{f}})$) in a single unbinding event, for different mean proofreading time. We set $k_{-1} = 1$ and $k'_{-1} = 2$.

rounds of binding and unbinding are required to achieve a high channel capacity.

A4 Multistep binding model

In the main text, we have considered the analysis of the multistep limit of the classical KPR model, where the processing time from initial binding to full activation is taken to be a deterministic time τ.

In the classical KPR model, the processing time is subject to noise due to finite number of steps in the activation process.

In this section, we fix the number of activation steps $m = 6$ and perform simulations using the same parameters as in the main text. The biophysical rationale derives from the six phosphorylation sites in the associated CD3 ζ chains. In particular, the phosphorylation kinetics of these sites are independent of the antigen types, except for two extra phosphorylation sites on ZAP70 that are regulated depending on the antigen types, which we do not explicitly consider in this paper. Figure A2 shows a qualitatively similar dependence of channel capacity on processing time τ as the deterministic limit in Fig 6. The channel capacity of product-based discrimination is maximized at $\tau \approx 0.5/k_1$, irrespective of T, while the optimal processing time for the channel capacity in a first activation time strategy increases with cell contact time T.

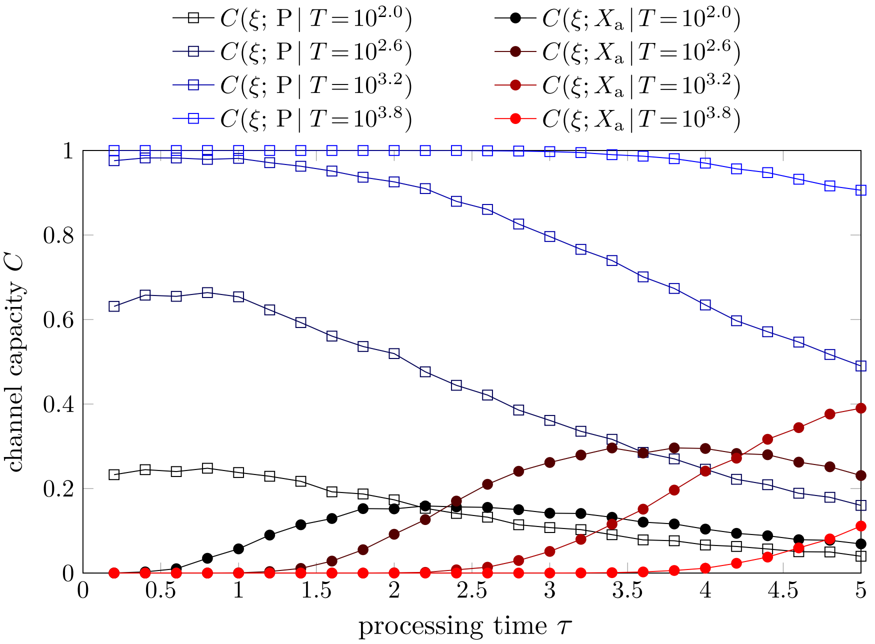

Fig A2. The channel capacities of product-based (blue squares) and first activation time-based (red dots) strategies as a function of processing time τ for various cell contact time T in a six-step multistep binding model. The parameter values are those used in Fig 6.

Similar dependences are observed for the channel capacity of a strategy based on whether protein numbers reach a threshold $\mathrm{P_{th}}$ within time T, as shown in Fig A3. These results are consistent with the behavior shown in Fig 9B. However, in both cases, the channel capacity of first-passage-time-based strategy with 6-step activation is lower than that of the deterministic limit, which is a consequence of the noise in the activation process. The channel capacity in the product-based discrimination strategy is similar to that of the deterministic limit, which may be explained by the buffering effect of product generation steps on upstream noise. In particular, decrease of $C(X_\mathrm{th}\,|\,\mathrm{P_{th}})$ is dependent on $\mathrm{P_{th}}$. Larger $\mathrm{P_{th}}$ leads to a smaller decrease of channel capacity. The optimal processing time for first-passage-time-based discrimination also increases, compared to the deterministic limit. A quantitative analysis awaits further investigation.

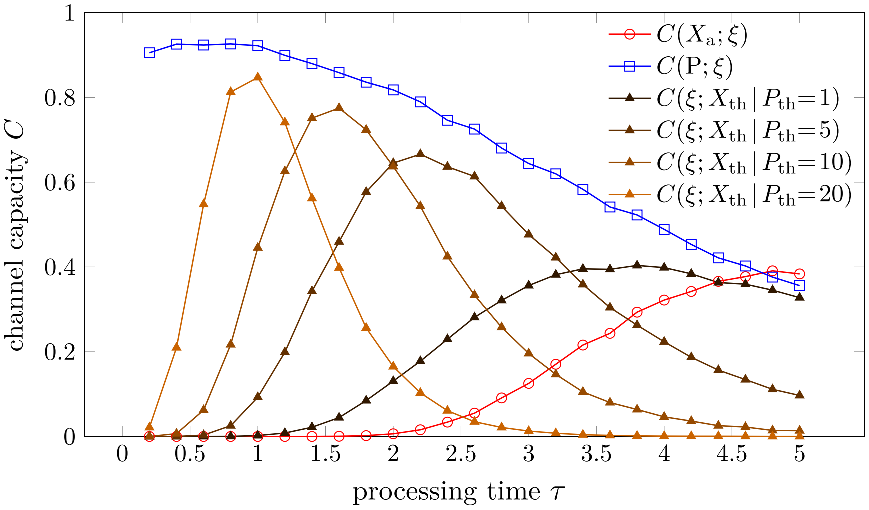

Fig A3. The channel capacity between the input ξ and the output X_{a}, $\mathrm{P}(T)$, or X_{th} as a function of processing time τ. The parameters used are the same as those in used in generating Fig 9. 10,000 independent Gillespie simulations are conducted for each τ.
